# Comparative effects of transcatheter versus surgical pulmonary valve replacement: A systematic review and meta-analysis

**DOI:** 10.1371/journal.pone.0322041

**Published:** 2025-05-20

**Authors:** Bunchai Chongmelaxme, Kok Pim Kua, Chanokpol Amornvetchayakul, Nichapond Chawviriyathep, Thunyapat Kerdklinhom

**Affiliations:** 1 Department of Social and Administrative Pharmacy, Faculty of Pharmaceutical Sciences, Chulalongkorn University, Bangkok, Thailand; 2 Revolution Health Research Consulting (RHRC) Group, Bangkok, Thailand; 3 Sultan Idris Shah Hospital, Serdang, Selangor, Malaysia; 4 A.S. Watson Group, Kuala Lumpur, Malaysia; 5 Stanford Alumni Association, Stanford University, Stanford, California, United States of America; 6 Harvard T.H. Chan School of Public Health, Harvard University, Boston, Massachusetts, United States of America; 7 MIT Alumni Association, Massachusetts Institute of Technology, Cambridge, Massachusetts, United States of America; Ataturk University Faculty of Medicine, TÜRKIYE

## Abstract

**Introduction:**

Transcatheter pulmonary valve replacement (TPVR) is developed as a non-surgical, minimally invasive procedure to reduce the need for re-do cardiac surgical interventions. However, its impacts on patient outcomes are less clear. This study aims to investigate the effects of TPVR among patients with pulmonary valve or right ventricular outflow tract dysfunctions.

**Materials and methods:**

In this systematic review and meta-analysis, we searched PubMed, Cochrane CENTRAL, EMBASE, CINAHL Complete, and Web of Science, from database inception to March 1, 2024, to identify studies that assessed the comparative effectiveness of transcatheter pulmonary valve replacement (TPVR) and surgical pulmonary valve replacement (SPVR). The key outcomes of interest included mortality, pulmonary regurgitation (PR), infective endocarditis (IE), re-intervention, improvements in cardiac failure based on the New York Heart Association (NYHA) functional classification, and adverse events. Meta-analyses using a random-effects model were performed.

**Results:**

A total of 28 studies (n = 16,150) were included. The meta-analyses depicted that when compared with SPVR, TPVR reduced risks of mortality by 36% (odds ratio [OR] = 0.64 [95% confidence interval, CI: 0.43, 0.95]), but conferred a three-fold greater odd of IE over the follow-up duration (OR = 3.10 [95% CI: 2.22, 4.33]). No significant differences were observed for 30-day mortality, and the early PR, IE and re-intervention, as well as the PR and re-intervention during follow-up. Meta-analyzed results across the outcome measures varied according to geographical region, publication year cut-off, and income status of country. All patients who had undergone valve replacement showed improvements in heart function and experienced relevant post-procedural complications.

**Conclusions:**

TPVR afforded significant clinical benefits in patient survival, but nonetheless, it was associated with an elevated risk for infective endocarditis.

## Introduction

Congenital heart disease (CHD) is a gross abnormality of the heart structure that is present at birth [1]. Certain defects are manageable and do not necessitate treatment, while others are more complicated and may require several surgeries performed sequentially over growing years [1,2]. Incidence of CHD has been reported to range from 5 to 8 per 1,000 live births [3,4]. CHD accounts for approximately one-third of all birth defects and afflicts over 13.3 million children globally [5]. An estimated 1 in 5 of patients with CHD suffer from pulmonary valve (PV) or right ventricular outflow tract (RVOT) dysfunctions, including defects, such as tetralogy of fallot (TOF), truncus arteriosus (TA), or pulmonary atresia (PA) [6]. The surgical repair during the first month of life is the current gold standard for treatment which is deemed to be potentially life-saving [7]. CHD accounts for approximately one-third of all birth defects and afflicts over 13.3 million children globally. The past few decades have seen some progress in interventional cardiology and congenital heart surgery, leading to a marked decrease in the worldwide mortality rate of CHD from 7.1 per 100,000 in 1990 to 2.8 per 100,000 in 2019 [5]. However, CHD remains a top cause of infant mortality, being ranked seventh globally and fourth in countries with a high socio-demographic index [8,9]. As the leading causes of global infant mortality continue to shift from communicable to non-communicable diseases with improving economies and public health systems [8], access to adequate and effective cardiac interventions and cardiovascular care is of crucial importance to enhance survival and improve quality of life and overall prognosis for people with CHD [9,10].

Advances in the technique of surgical pulmonary valve replacement (SPVR) have been less promising. Patients undertaking SPVR have limited longevity of bioprosthetic valves and conduits and are prone to progressive RVOT impairment owing to progressive calcification of heart valves. This leads to pulmonic stenosis (PS) and/or pulmonary regurgitation (PR), right ventricular dysplasia, arrhythmia, and death if left untreated in the long term [11]. PS is narrowing, obstruction, or stiffening of the pulmonary valve commonly associated with congenital structural cardiac syndromes, such as TOF and Noonan syndrome. In adults, PS occurs during pregnancy and among those with underlying carcinoid syndrome, rheumatic heart disease, prior cardiothoracic surgeries, or a cardiac tumor [12]. PR is the leakage of pulmonary valve that causes backflow of blood into the right ventricle. Predominant etiologies of PR encompass pulmonary hypertension and congenital heart defects, particularly TOF. Less common causes of PR include surgical valvotomy, vulvectomy or balloon pulmonary valvuloplasty, rheumatic heart disease, infective endocarditis, carcinoid syndrome, and ingestion of medications that affect serotoninergic pathways [13]. Numerous re-do cardiac surgeries are hence warranted across the patients’ lifespan with accompanying risks of perioperative complications and mortality [7,11]. Transcatheter pulmonary valve replacement (TPVR) was originally designed as a non-surgical, minimally invasive method to decrease the frequency of re-do surgical operations among patients who had PS and/or PR in an existing right ventricle-pulmonary artery conduit and such application was then extended more broadly to include patient with native right ventricular outflow tract. Yet, patients treated with TPVR do overwhelmingly bear the risks of infective endocarditis (IE), re-intervention, as well as mortality [14,15].

Although several existing systematic reviews and meta-analyses had examined the clinical effects of TPVR versus SPVR [16–18], the overall impact of TPVR remained less clear due to inconsistent findings, on top of several new studies being published after the latest review. Therefore, the current study aims to update the evidence and provide the latest high-quality insights into the effects of TPVR to inform clinical decisions or facilitate future implementation by healthcare professionals and policy makers.

## Materials and methods

### Search strategy

An electronic literature search was performed from inception to March 1, 2024, on the following databases: PubMed, Cochrane CENTRAL, EMBASE, CINAHL Complete, and Web of Science. All the search terms are presented in S1 Table. The bibliographies of retrieved articles were also examined to identify relevant studies that were not indexed in the databases.

### Study selection

Initially, the titles and abstracts were screened by CA, NC, and TK to identify potential studies. Studies were included within the systematic review if they were clinical trials, cohort studies or case-control studies that investigated the effects of TPVR compared with SPVR among adult or pediatric patients with congenital heart disease of malfunctioning PV or RVOT. Full texts of potentially relevant studies were assessed by CA, NC, and TK and all disagreements between investigators were resolved by consensus with independent reviewers (BC and KP).

### Data extraction, definition of outcomes and quality assessment

Data abstraction was undertaken by CA, NC, and TK with the use of a standardized template. The extracted data encompassed the author’s names, publication years, country of study, study designs, the characteristics of participants, interventions, comparators, and study outcomes. Outcomes of interest were mortality, PR, IE, re-intervention, improvements in cardiac impairment, and adverse events. Mortality was defined as the number of patient deaths after receiving the procedure, whereas PR was the presence of any significant manifestation with at least the grade of moderate. IE was the reported cases after receiving procedures and re-intervention was defined as re-intervention or re-operation, including re-opening, re-catheterization, surgical explantation, or conduit replacement. The improvements in cardiac impairment were evaluated based on the New York Heart Association (NYHA) functional classification [19] and the adverse events were documented from all events reported in the individual studies. Only the mortality outcomes were recorded within 30 days or over the study follow-up period (after 30 days), whereas the other outcomes were categorized as early (within 6 months) or throughout the length of follow-up (after 6 months). All the included studies were assessed for their methodological qualities using the Cochrane risk of bias tool for randomized-controlled trials (RCTs) [20] and the Newcastle-Ottawa scale [21] for cohort and case-control studies. Our study protocol was registered in the PROSPERO database (CRD42022314221).

### Data analysis

A meta-analysis was performed to estimate the effects of TPVR compared with SPVR using the odds ratio (OR) along with 95% confidence interval (CI). The DerSimonian and Laird random-effects models were employed to account for variability within- and between- studies, where heterogeneity among studies was measured using *I*^2^ and Chi-square (χ^2^) statistical tests [20]. To add novelty for meta-analysis findings, all the outcome analyses were carried out based on 3 principal domains of the included studies: 1) Geographical region (East Asia & Pacific vs Europe & Central Asia vs Middle East & North Africa vs North America), 2) Year of publication (prior to 2019 vs post 2019), and 3) Country income level (low-and-middle-income vs high-income). Potential sources of heterogeneity were explored using subgroup technique accordingly. Additionally, a net clinical benefit analysis was performed to quantify the risk and benefit of significant findings [22]; the risk difference (RD) alongside 95% CI was estimated for each study and pooled across studies using random-effects model and subsequently computing the incremental risk and benefit ratio (IRBR). The data in relation to incremental risk and incremental benefit were further simulated using a Monte Carlo simulation with 1,000 iterations with an assumed normal distribution for both. The results were presented in a cost-effective plane by assigning incremental risk on the Y-axis and incremental benefit on the X-axis, at varying threshold levels and represented as 95% credible interval (CrI). All analyses were performed using STATA version 14 and Microsoft Excel 2016.

## Results

**Fig 1:**
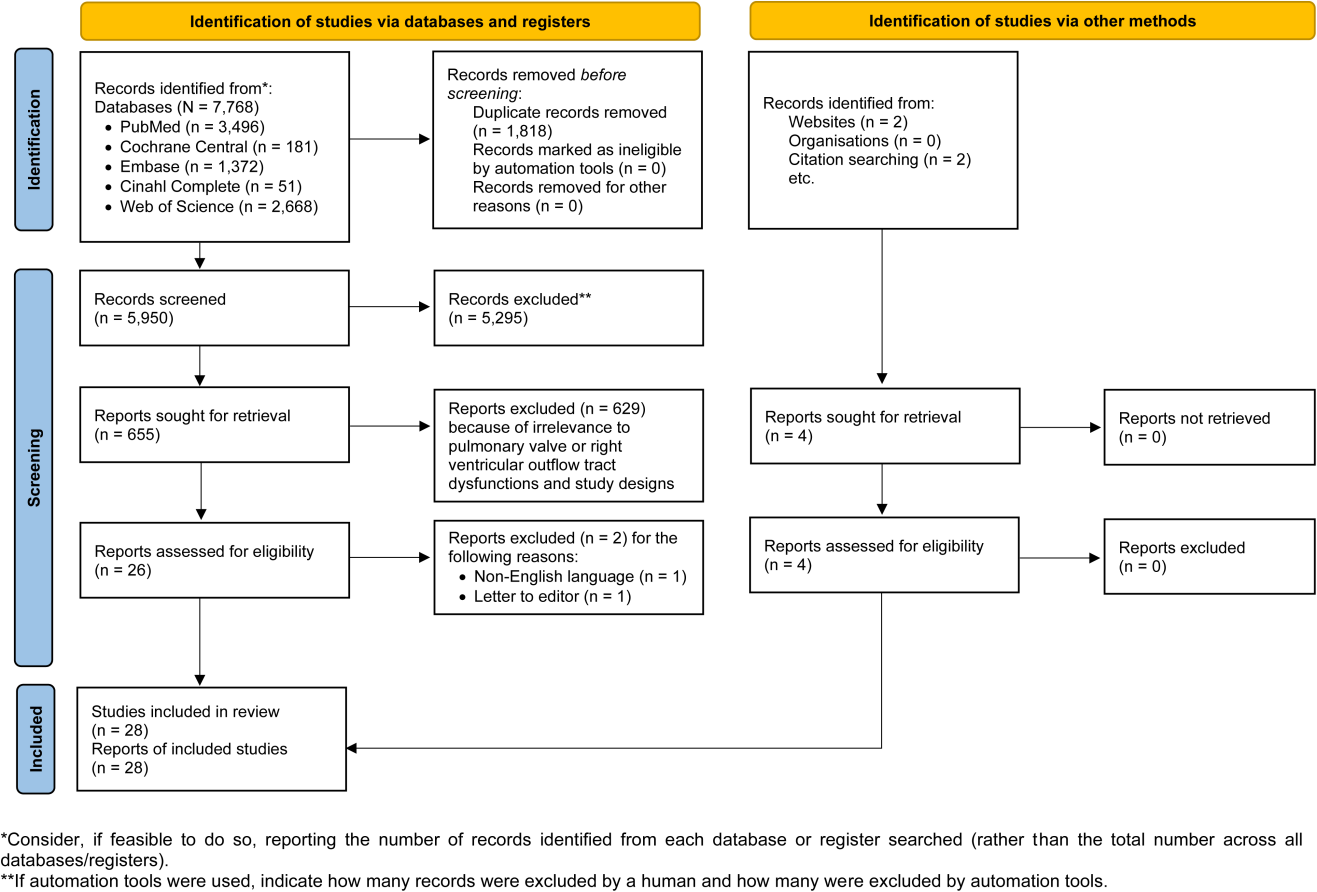
A PRISMA flow diagram describes the study selection process.

The initial search yielded 7,772 articles, of which 1,818 duplicates were removed. The remaining 5,950 articles were screened on the basis of titles and abstracts. A total of 629 were excluded because of their irrelevance to PV or RVOT dysfunctions and the study designs. Together with 4 articles identified from websites or citation searching, we had 28 full-text articles included in the final qualitative and quantitative syntheses. The Preferred Reporting Items for Systematic Reviews and Meta-Analyses (PRISMA) flow diagram is depicted in Fig 1. The results of the database search are presented in S1 Table.

### Study characteristics

Among 28 included studies, 15 were from the US [23–37]. Each of the 2 studies was from Germany [38,39], Denmark [40,41], and the UK respectively [42,43]. The others were from China [44], France [45], New Zealand [46], Norway [47], Saudi Arabia [48], Sweden [49], and Thailand [50]. All were retrospective cohort in design, except only one was a prospective case-control study [47]. A total of 3,650 patients were enrolled in the TPVR group, while the SPVR group had 12,500 patients. Mean age of the patients in TPVR and SPVR groups ranged from 14 to 36 years [43,47] and 7–33 years respectively [39,40]. Follow-up durations varied from 2.4 months to 10.4 years [39,41]. The most frequently used of TPVR approaches were Melody (11 studies) [24,30–33,35,38,40,45,46,48], followed by the combination of Melody and SAPIEN (8 studies) [23,26,27,37,39,41,43,47]. One study deployed Venus P-valve [44] and another study utilized a combination of Melody, Pulsta, SAPIEN, and Venus P-valves [50]. Another 7 studies did not report specific details of TPVR used [25,28,29,34,36,42,49]. For SPVR, 2 studies applied Contegra and Homograft [33,40], whilst 1 study employed Homograft [44] and one study each used a combination of Contegra, Hancock and Homograft [39], Contegra, Hancock, Homograft, and others [38], Contegra, Homograft and Perimount Magna [47], Contegra, Freestyle bioprosthesis, Homograft and Perimount Magna [50], and Contegra, Homograft, Mosaic, Perimount Magna, and Trifecta [23]. The others (19 studies) did not report the exact types of SPVR being used (Table 1) [24–32,34–37,42,43,45,46,48,49].

**Table 1:** Characteristics of the included studies.

First Author (year)	Country	Settings	Patient Characteristics		Sample Size (TPVR vs SPVR)	Trade Name		Follow-Up Duration (Months)
			Age (y)	Male (n, %)		TPVR	SPVR	
Alassas (2018) [48]	Saudi Arabia	King Faisal Specialist Hospital & Research Center	TPVR = 24 ± 10 SPVR = 27 ± 11	TPVR = 39 (83) SPVR = 26 (63)	47 vs 41	Melody	NR	TPVR = 56.0 ± 24.0 SPVR = 89.0 ± 46.0
Andresen (2018) [47]	Norway	Oslo University Hospital	TPVR = 14 (range: 8, 36) SPVR = 23 (range: 9, 53)	TPVR = 13 (65.0) SPVR = 9 (64.5)	20 vs 14	1) Melody 2) SAPIEN	1) Contegra 2) Homograft 3) Perimount Magna	12
Bou Chaaya (2023) [36]	US	Riley Children’s Hospital, Indianapolis Houston Methodist DeBakey Heart and Vascular Center	TPVR = 12.7 (IQR 6.0, 24.0) SPVR = 17.0 (IQR 8.4, 36.0)	TPVR = 12 (44) SPVR = 30 (51)	27 vs. 58	NR	NR	43.2 (IQR: 26.4, 74.4)
Caughron (2018) [23]	US	Emory Healthcare	TPVR = 30.5 (IQR: 24.3, 35.0) SPVR = 29.5 (IQR: 23.0, 44.3)	TPVR = 13 (36.1) SPVR = 19 (63.3)	36 vs 30	1) Melody 2) SAPIEN	1) Contegra 2) Homograft 3) Mosaic/Hancock 4) Perimount Magna 5) Trifecta	25.9 (IQR: 12.25, 46.45)
Coats (2005) [42]	UK	Great Ormond Street Hospital for Children	TPVR = 25 (range: 6, 65) SPVR = 16 (range: 9, 38)	TPVR = 16 (65.7) SPVR = 63 (67.0)	35 vs 94	NR	NR	TPVR = 4.0 (range: 0.1, 59.5) SPVR = 10.0 (range: 0.1, 14.3)
Daily (2018) [24]	US	The Pediatric Health Information Systems Database	TPVR = 17 (IQR: 13, 24) SPVR = 16 (IQR: 12, 22)	TPVR = 110 (58) SPVR = 236 (62)	191 vs 382	Melody	NR	NR
Durongpisitkul (2022) [50]	Thailand	Faculty of Medicine Siriraj Hospital Mahidol University	TPVR = 20.7 (IQR: 17.3, 26.2) SPVR = 28.2 (IQR: 20.7, 42.4)	TPVR = 44 (61.1) SPVR = 75 (52.4)	72 vs 143	1) Melody 2) Pulsta 3) SAPIEN 4) Venus P-valve	1) Contegra 2) Freestyle bioprosthesis 3) Homograft 4) Perimount Magna	24
Egbe (2024) [37]	US	Mayo Clinic Rochester	TPVR = 36 ± 14 SPVR = 33 ± 11	TPVR = 34 (53) SPVR = 68 (53)	64 vs. 128	1) Melody 2) SAPIEN	NR	108
Enezate (2019) [25]	US	The Nationwide Readmissions Database	TPVR = 25 ± 14.3 SPVR = 26.4 ± 18.6	TPVR = 98 (55.7) SPVR = 474 (59.3)	176 vs 799	NR	NR	NR
Georgiev (2020) [38]	Germany	German Heart Center	TPVR = 19 (range: 5, 79) SPVR = 18 (range: 5, 65)	NR	241 vs 211	Melody	1) Contegra 2) Hancock 3) Homograft 4) Others	TPVR = 57.6 (range: 2.4, 139.2) SPVR = 76.8 (range: 2.4, 151.2)
Gröning (2019) [40]	Denmark	Rigshospitalet	TPVR - Melody = 20 (IQR: 14, 33) SPVR - Contegra = 7 (IQR: 1, 14) - Homograph = 20 (IQR: 7, 41)	TPVR = 42 (66) SPVR = 204 (55)	64 vs 368	Melody	1) Contegra 2) Homograft	TPVR - Melody = 46.8 (IQR: 1.0, 6.8) SPVR - Contegra = 72.0 (IQR: 38.4, 98.4) - Homograph = 8.3 (IQR: 3.6, 13.1)
Gröning (2024) [41]	Denmark	NR	NR	NR	83 vs. 463	1) Melody 2) SAPIEN	1) Homograft 2) Contegra 3) Perimount/Magna 4) Magna Ease 5) Hancock 6) Freestyle	124.8 (IQR: 43.2, 198)
Haas (2018) [39]	Germany	Medical Hospital of the University of Munich	TPVR - Melody = 13.0 ± 4.0 - SAPIEN = 20.1 ± 9.5 SPVR - Contegra = 8.1 ± 5.8 - Hancock = 33.3 ± 24.0 - Homograft = 26.2 ± 15.6	TPVR = 47 (58.8) SPVR = 98 (59.0)	80 vs 166	1) Melody 2) SAPIEN	1) Contegra 2) Hancock 3) Homograft	TPVR - Melody = 51.6 - SAPIEN = 28.8 SPVR - Contegra = 37.2 - Hancock = 2.4 - Homograft = 20.0
Hribernik (2022) [43]	UK	Leeds Congenital Heart Centre	TPVR - Melody = 24 (range: 5, 64) - SAPIEN = 36 (range: 12, 76) SPVR = 23 (range: 0.2, 65)	TPVR = 73 (60.8) SPVR = 225 (61.6)	120 vs 365	1) Melody 2) SAPIEN	NR	TPVR = 17 (range: 0, 116) SPVR = 47 (range: 0, 243)
Li (2017) [35]	US	Yale New Haven Children’s hospital	TPVR = 22.1 (range: 4.7, 55.3) SPVR = 18 (range: 5, 65)	TPVR = 18 (55) SPVR = 16 (52)	32 vs 30	Melody	NR	TPVR = 24.8 (range: 7.1, 46.6) SPVR = 23.2 (range: 7.4, 49.4)
Lluri (2018) [26]	US	University of California, Los Angeles Ronald Reagan Medical Center	TPVR = 25.7 ± 15.2 SPVR = 23.3 ± 16.1	TPVR = 118 (57) SPVR = 72 (54)	208 vs 134	1) Melody 2) SAPIEN	NR	TPVR = 26.4 (IQR: 1.0, 3.1) SPVR = 33.6 (IQR: 0.9, 4.0)
Malekzadeh-Milani (2014) [45]	France	Necker Hospital for Sick Children	TPVR = 20.1 (95% CI: 18.2, 21.8) SPVR = 13.3 (95% CI: 11.6, 15.0)	TPVR = 52 (55.9) SPVR = 111 (56.9)	93 vs 195	Melody	NR	TPVR = 23.8 (95% CI: 17.5, 32.5) SPVR = 24.1 (95% CI: 19.9, 29.9)
Megaly (2021) [27]	US	The National Inpatient Sample, Health Cost and Utilization Project, and Agency for Healthcare Research and Quality	TPVR = 21 (IQR: 14, 35) SPVR = 17 (IQR: 10, 33)	TPVR = 700 (61.4) SPVR = 2505 (58.2)	1140 vs 4305	1) Melody 2) SAPIEN	NR	NR
O’Byrne (2015) [28]	US	The Pediatric Health Information Systems Database	TPVR = 20.5 (IQR: 10, 64) SPVR = 17 (IQR: 10, 61)	TPVR = 15 (50) SPVR = 436 (57)	30 vs 769	NR	NR	NR
O’Byrne (2016) [29]	US	The Pediatric Health Information Systems Database	TPVR = 17.7 (IQR: 13.1, 24.8) SPVR = 16.2 (IQR: 12.5, 21.3)	TPVR = 180 (62) SPVR = 1113 (61)	292 vs 1816	NR	NR	NR
O’Donnell (2017) [46]	New Zealand	Green Lane Pediatric and Congenital Cardiac Service	TPVR = 18 (range: 11, 51) SPVR = 16 (range: 0, 61)	TPVR = 15 (60.0) SPVR = 112 (62.9)	25 vs 178	Melody	NR	35.5 (range: 3, 67)
Ou-Yang (2020) [44]	China	Fuwai Hospital, Shanghai Hospital, and West China Hospital	TPVR = 30.1 ± 12.2 SPVR = 25.1 ± 12.3	TPVR = 11 (31.4) SPVR = 13 (43.3)	35 vs 30	Venus P-valve	Homograft	TPVR = 36 (IQR: 36, 48) SPVR = 36 (IQR: 33, 48)
Sharma (2018) [30]	US	Primary Children’s Hospital	TPVR = 19 ± 13 SPVR = 12 ± 7	TPVR = 71 (58) SPVR = 54 (54)	124 vs 100	Melody	NR	TPVR = 18.7 ± 17.0 SPVR = 31.6 ± 22.0
Skoglund (2017) [49]	Sweden	Swedish National Registry for Congenital Heart Disease	32.3 (range: 18, 71)	332 (58)	50 vs 762	NR	NR	190.8 (95% CI: 181.2, 200.4)
Sosnowski (2016) [31]	US	Rush University Medical Center	TPVR = 31.5 ± 7.43 SPVR = 31.3 ± 18.37	TPVR = 4 (50.0) SPVR = 8 (61.5)	8 vs 13	Melody	NR	TPVR = 3.4 ± 4.58 SPVR = 13.6 ± 11.98
Steinberg (2017) [32]	US	Seattle Children’s Hospital University of Washington Medical Center	TPVR = 25.9 ± 1.6 SPVR = 25.8 ± 1.2	TPVR = 45 (57.7) SPVR = 76 (52.4)	78 vs 145	Melody	NR	NR
Van Dijck (2014) [33]	US	University Hospitals Leuven	TPVR - Melody = 14.3 (range: 4, 80) SPVR - Contegra = 9.9 (range: 0, 47) - Homograft = 13.3 (range: 0, 60)	TPVR = 71 (66.4) SPVR = 384 (60.9)	107 vs 631	Melody	1) Contegra 2) Homograft	TPVR - Melody = 24.0 SPVR - Contegra = 78.0 - Homograft = 105.6
Wadia (2018) [34]	US	University of California, Los Angeles Medical Center	TPVR = 21.7 (IQR: 15.6, 30.9) SPVR = 21.0 (IQR: 14.2, 35.9)	TPVR = 107 (62) SPVR = 69 (53)	172 vs 130	NR	NR	TPVR = 15.6 (IQR: 2.4, 33.6) SPVR = 12.0 (IQR: 1.2, 34.8)

IQR, interquartile range; NR, no report; SPVR, surgical pulmonary valve replacement; TPVR, transcatheter pulmonary valve replacement.

### Meta-analysis

#### Mortality.

**Fig 2:**
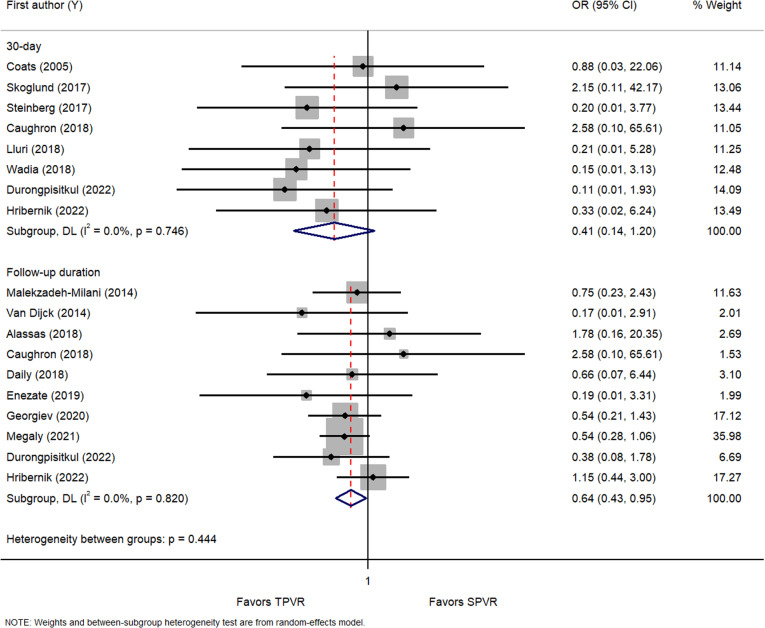
Forest plot of 30-day mortality and the mortality during follow-up. OR, odds ratio.

The effects of TPVR on 30-day mortality were demonstrated in 9 studies [23,26,32,34,42,43,48–50] and 16 studies [23–25,27–31,33,38,43–45,47,48,50] reported mortality throughout the study follow-up period (S2 Table). TPVR depicted a non-significant difference when compared with SPVR in 30-day mortality (OR = 0.41 [95% CI: 0.14, 1.20], *I*^2^ = 0.0%, *P* = 0.746), however, it could reduce the mortality during follow-up by 36% (OR = 0.64 [95% CI: 0.43, 0.95], *I*^2^ = 0.0%, *P* = 0.820) (Fig 2).

In addition, TPVR depicted a non-significant difference when compared with SPVR in 30-day mortality across East Asia & Pacific (OR = 0.11 [95% CI: 0.01, 1.93]), Europe & Central Asia (OR = 0.85 [95% CI: 0.15, 4.88], *I*^2^ = 0.0%, *P* = 0.682), and North America (OR = 0.34 [95% CI: 0.07, 1.59], *I*^2^ = 0.0%, *P* = 0.574), however, it could reduce the mortality during follow-up only in North America by 47% (OR = 0.53 [95% CI: 0.29, 0.96], *I*^2^ = 0.0% *P* = 0.728) (S1, S2 Figs). We found that TPVR and SPVR had similar effects on 30-day mortality for studies published prior to 2019 (OR = 0.55 [95% CI: 0.15, 1.93], *I*^2^ = 0.0% *P* = 0.660) and after 2019 (OR = 0.19 [95% CI: 0.02, 1.47], *I*^2^ = 0.0% *P* = 0.596), while there was a reduction in risk of 39% for mortality during follow up in TPVR studies published after 2019 (OR = 0.61 [95% CI: 0.39, 0.95], *I*^2^ = 0.0% *P* = 0.589) (S3, S4 Figs). In terms of country income level, no significant differences in outcomes of 30-day mortality and mortality during follow-up were observed between TPVR and SPVR patient groups (S5, S6 Figs).

#### Pulmonary regurgitation.

**Fig 3:**
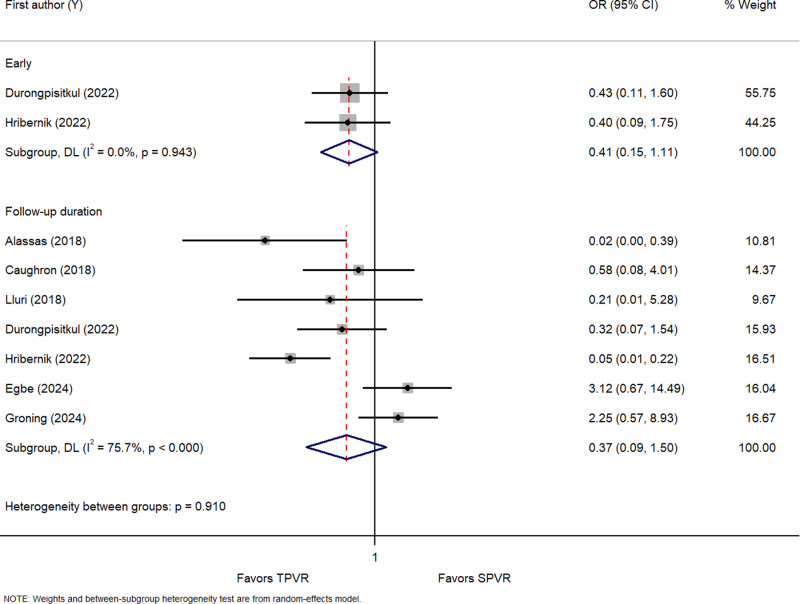
Forest plot of the early and pulmonary regurgitation during follow-up. OR, odds ratio.

Three studies [23,43,50] and eight studies [23,26,31,37,41,43,48,50] investigated the effects of TPVR on early PR and the PR during follow-up, respectively (S3 Table). Compared with SPVR, TPVR had no marked difference in early PR (OR = 0.41 [95% CI: 0.15, 1.11], *I*^2^ = 0.0%*, P* = 0.943) and PR during follow-up (OR = 0.37 [95% CI: 0.09, 1.50], *I*^2^ = 75.7%*, P* < 0.000) (Fig 3).

Compared with SPVR, TPVR had no marked difference in early PR in East Asia & Pacific (OR = 0.43 [95% CI: 0.11, 1.60]) and Europe & Central Asia (OR = 0.40 [95% CI: 0.09, 1.75], while it was found to reduce the PR during follow-up only in Middle East & North Africa by 98% (OR = 0.02 [95% CI: 0.00, 0.39]) (S7, S8 Figs). TPVR showed a non-significant difference when compared to SPVR for the early PR and PR during follow-up in studies published prior to 2019 and post 2019 (S9, S10 Figs). Likewise, it showed a non-significant difference among different country income levels (S11, S12 Figs).

#### Infective endocarditis.

**Fig 4:**
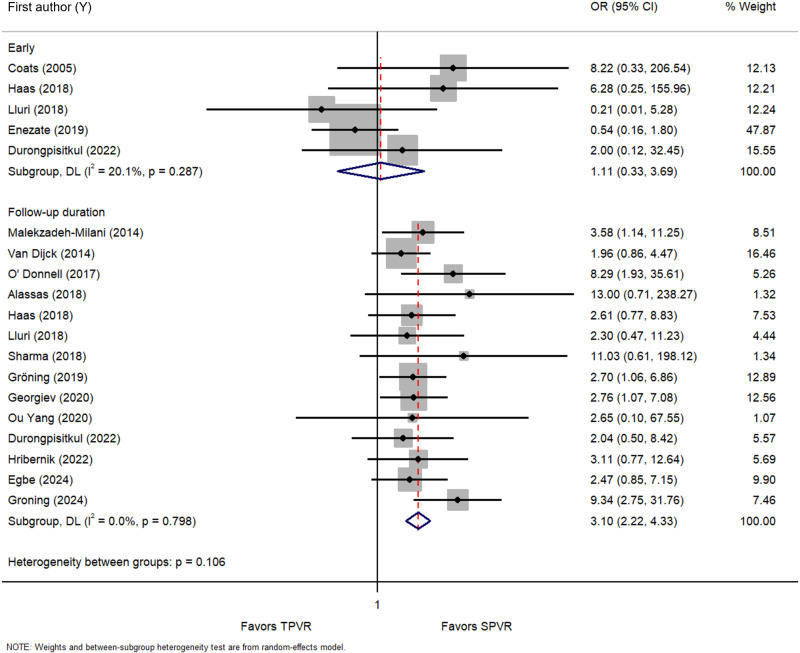
Forest plot of the early and infective endocarditis during follow-up. OR, odds ratio.

Six studies [23,25,26,39,42,50] and fifteen studies [23,26,30,33,37–41,43–48] examined the effects of TPVR on early IE and over the follow-up duration, respectively (S4 Table). TPVR showed no discernible differences when compared with SPVR in terms of early IE (OR = 1.11 [95% CI: 0.33, 3.69], *I*^2^ = 20.1%, *P* = 0.287), nevertheless, it increased the risk of IE during follow-up by 3.10 times (OR = 3.10 [95% CI: 2.22, 4.33], *I*^2^ = 0.0%, *P* = 0.798) (Fig 4).

TPVR showed no discernible differences when compared with SPVR in terms of early IE; East Asia & Pacific (OR = 2.00 [95% CI: 0.12, 32.45]), Europe & Central Asia (OR = 7.18 [95% CI: 0.74, 69.89], *I*^2^ = 0.0%, *P* = 0.908) and North America (OR = 0.48 [95% CI: 0.15, 1.48], *I*^2^ = 0.0%, *P* = 0.598), nevertheless, it increased the risk of IE during follow-up across the regions; East Asia & Pacific (OR = 3.88 [95% CI: 1.47, 10.24], *I*^2^ = 0.0%, *P* = 0.391), Europe & Central Asia (OR = 3.39 [95% CI: 2.16, 5.33], *I*^2^ = 0.0%, *P* = 0.662) and North America (OR = 2.31 [95% CI: 1.28, 4.17], *I*^2^ = 0.0%, *P* = 0.731), except Middle East and North Africa (OR = 13.00 [95% CI: 0.71, 238.27) (S13, S14 Figs). TPVR also exhibited a non-significant difference in early IE for the period prior to 2019 and post 2019 as compared with SPVR, while it could increase IE during follow up in prior to 2019 by 3.09-fold (OR = 3.09 [95% CI: 1.87, 5.08], *I*^2^ = 0.0% *P* = 0.559) and post 2019 by 3.12-fold (OR = 3.12 [95% CI: 1.99, 4.89], *I*^2^ = 0.0% *P* = 0.706) (S15,S16 Figs). In terms of country income levels, non-significant differences were observed for early IE, but there was an increased risk of IE during follow up in high-income nations (OR = 3.19 [95% CI: 2.25, 4.50], *I*^2^ = 0.0% *P* = 0.686) (S17, S18 Figs).

#### Re-intervention.

**Fig 5:**
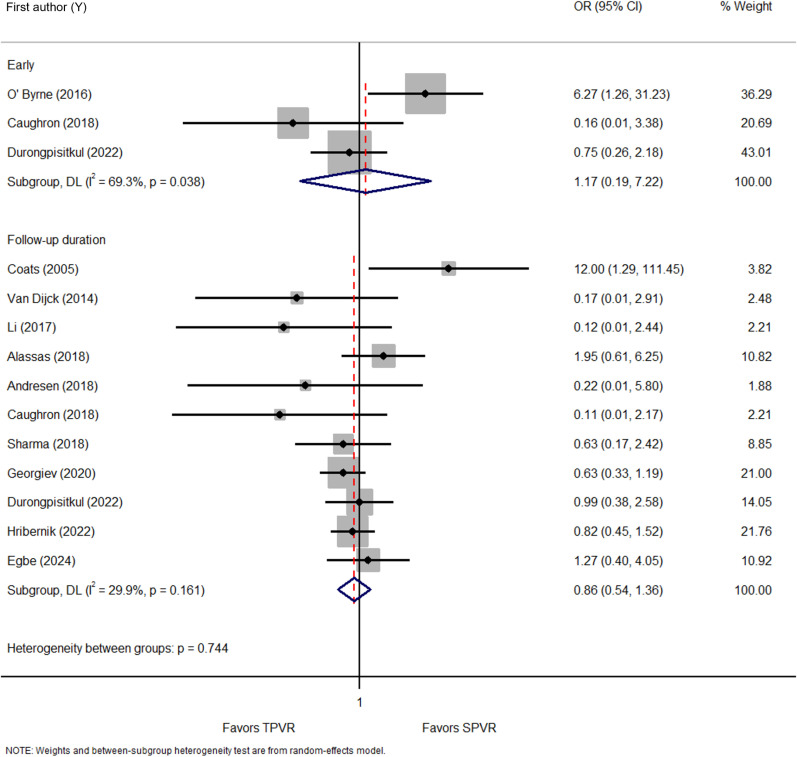
Forest plot of early re-intervention and the re-intervention during follow-up. OR, odds ratio.

Three studies [23,29,50] and eleven studies [23,30,33,35,37,38,42,43,47,48,50] evaluated the effects of TPVR on early re-intervention and over the follow-up duration, respectively (S5 Table). TPVR appeared to be inferior to SPVR in the early re-intervention outcome (OR = 1.17 [95% CI: 0.19, 7.22], *I*^2^ = 69.3%*, P* = 0.038) and had similar risk of re-intervention over the follow-up duration (OR = 0.86 [95% CI: 0.54, 1.36], *I*^2^ = 29.9%*, P* = 0.161) (Fig 5).

TPVR demonstrated a similar risk of re-intervention across regions during the early phase; East Asia & Pacific (OR = 0.75 [95% CI: 0.26, 2.18] and North America (OR = 1.26 [95% CI: 0.03, 45.61], *I*^2^ = 77.0%, *P* = 0.037) and over the duration of follow-up; East Asia & Pacific (OR = 0.99 [95% CI: 0.38, 2.58]), Europe & Central Asia (OR = 0.92 [95% CI: 0.40, 2.12], *I*^2^ = 56.0%, *P* = 0.078), Middle East & North Africa (OR = 1.95 [0.61, 6.25]) and North America (OR = 0.56 [95% CI: 0.23, 1.37], *I*^2^ = 14.8%, *P* = 0.320) (S19, S20 Figs). TPVR showed a non-significant difference as compared to SPVR for early re-intervention and over the follow-up duration across years of publication (S21, S22 Figs) and country income levels (S23, S24 Figs).

Given the heterogeneity in the definitions of outcome measures, studies that assessed improvements in cardiac impairment (4 studies) [23,26,43,44] and adverse events (14 studies) [23,25,27,30,32,34,39,42–44,46–48,50] were excluded from the meta-analysis to prevent potential bias in pooled estimates. In brief, improved cardiac impairment was noted in 3 studies [23,26,43] for the early phase and 4 studies [23,26,43,44] for the follow-up duration (S6 Table). A study by *Caughron*, et al [23] reported that the proportion of patients with TPVR remained in NYHA functional class 3–4 was decreased from 75% (at baseline) to 2.8% (at early) and 0% (over the duration of follow-up), while those with SPVR were reduced from 33.3% to 3.3% and 0%, respectively. A study by *Lluri*, et al [26] reported that the percentage of patients with TPVR who remained in class 1 was increased from 27% (at baseline) to 81% (at early and over follow-up duration), while those with SPVR were increased from 31% to 87%. A study by *Hribernik*, et al [43] revealed a different pattern of percentages of patients with TPVR across each functional class (from 1 to 4) that were 39.8%, 33.6%, 21.9% and 4.7% (at baseline) to 75.9%, 22.3%, 0.9% and 0.9% (at early) and 74.8%, 18.9%, 5.4% and 0.9% (over the duration of follow-up) and for those with SPVR were 53%, 36%, 9.3% and 1.7% (at baseline) to 84.1%, 14.3%, 0.8% and 0.8% (at early) and 86.9%, 8.4%, 2.5% and 2.2% (over the duration of follow-up). Lastly, a study by *Ou-Yang*, et al [44] documented that the proportions of patients with TPVR in each class (from 1 to 3) changed from 2.9%, 77.1% and 20% (at baseline) to 94.3%, 5.7% and 0% (over the duration of follow-up) and those with SPVR from 13.3%, 80.0% and 6.4% (at baseline) to 93.9%, 6.7% and 0% (over the duration of follow-up).

Among 17 studies [23,25,27,30,32,34,36,37,39,41–44,46–48,50] reporting the adverse events (S7 Table), the most frequently observations documented from the patients with TPVR were cardiac complications (42.4%) [39], followed by pulmonary stenosis (28.6%) [41], prosthetic valve dysfunction (17.2%) [37], late RVOT restenosis (17.1%) [42], non-intraoperative postprocedural events that affected patient comfort, functional status or length of hospitalization (12.8%) [34], ventricular arrhythmia (12.3%) [27], all major complications that necessitated cardiopulmonary resuscitation and prolonged ICU or hospital stays (11.1%) [50] and so forth. For those with SPVR, pulmonary stenosis (64.9%) [41], all major complications required cardiopulmonary resuscitation and prolonged ICU or hospital admissions (32.9%) [50], non-intraoperative postprocedural events that impacted patient comfort, functional status, or length of hospitalization (32.3%) [34] were the most frequently reported events, followed by cardiac complications (29.1%) [39], bleeding (26.4%) [25], respiratory complications (23.3%) [25], and others.

#### Risk and benefit assessment.

The results of pooled RD for incremental risk (IE) and incremental benefits (survival) are demonstrated in S25, S26 Figs. Compared with IE, the IRBRs of survival was approximately 4.22 (95% CrI: 2.05, 12.32) (S8 Table). The development of IE was approximately 4 times higher compared to survival. A Monte Carlo simulation with 1,000 iterations illuminated that if the threshold was set at 0.5, use of TPVR would not gain benefits from survival (S27 Fig).

#### Study quality assessment.

The Newcastle-Ottawa Scale was applied to assess the risk of bias in cohort [23–35,38–40,42–46,48–50] and case-control studies [47] and the results showed that most studies (15 studies) [25–28,30,32,33,39,40,42,43,45,46,49,50] were of poor quality, whilst another 12 studies [23,24,29,31,34–38,41,44,48] judged to have good quality. Only one study was rated to be fair (S9 Table) [42].

## Discussion

This study provided a comprehensive synthesis of the best available evidence regarding the effects of TPVR compared with SPVR among the CHD patients with PV and RVOT dysfunctions. Our findings revealed that TPVR reduced the risks of mortality, but it was associated with an elevated risk of IE. All patients undergoing valve replacement improved their cardiac impairment and experienced related complications. We further explored the net clinical benefit by considering significant risk and benefit simultaneously and found that patients receiving TPVR would experience IE for about 4 times higher than those who survived and there was no clear survival benefit with TPVR intervention.

The findings from a previous review by *Zhou* et al [16] highlighted that TPVR was able to reduce the risk of mortality during follow-up compared to SPVR, but had no benefit in terms of 30-day mortality. Likewise, another review by *Ribeiro* et al [17] suggested no differences in 30-day mortality and mortality during follow-up among patients under-replaced their valve in both groups. Both existing publications showed a significant reduction in PR risk. In comparison to the review by *Zhou* et al [16], our findings concurred and similarly suggested the roles of TPVR in reducing mortality during follow-up. TPVR reduces the number of repeated sternotomies and offers a minimally invasive technique, while the patients do not require cardiopulmonary bypass support during the operation that could prevent potential unfavorable events, such as ischemia reperfusion injury and inflammatory, arrhythmogenic effect, and so on, thus resulting in a lower risk of developing complications, an accelerated time to recovery, a shorter duration of hospital stay, and smaller incisions or scars [31,34]. In addition, TPVR was found to improve ventricular functions in right and left ventricles, where hemodynamics including PR fraction and RVOT gradient were also decreased over time [51]. Although TPVR was not considerably different from SPVR in modifying 30-day mortality and development of PR, a plausible explanation for this finding is that all patients received appropriate and well postoperative care and management algorithms from the healthcare providers, thus leading to low event rates. Furthermore, biomedical technology and hospital equipment are continuously being developed to offer tailored or more personalized care for patients who underwent valve replacement could diminish the occurrence of any unfavorable events and save lives, thus contributing to enhanced quality of healthcare services and life outcomes [52].

In contrast to our findings, the systematic review and meta-analysis by *Ribeiro* et al [17] indicated a non-significant difference in 30-day mortality and the mortality during follow-up among patients who received either TPVR or SPVR. *Ribeiro* et al included only 7 studies for 30-day mortality and 4 studies for the mortality during follow-up in their analyses [17], whereas our current review included 8 and 10 studies for 30-day mortality and that during follow-up, respectively. A study by *Dilber* et al [53] was not contained within our review because it was classified as a letter to the editor that did not fit our study’s eligibility criteria. Including the *Dilber* et al study [53] in our meta-analyses would also induce methodological heterogeneity in light of the variation in the included study design, which affects the quality of pooled estimates. We believe that the current systematic review and meta-analysis generates the most updated evidence pertaining to the effects of TPVR based on justified assumptions and study methodology. Notably, we observed a 39% reduced risk of mortality during follow up in studies published after 2019 and 98% reduced risk of PR during follow-up in Middle East & North Africa regions. A larger sample size would be desirable to provide more accurate values with smaller margins of error and lower standards of deviation, thus increasing reliability and validity of the findings. It is also noteworthy that technology continues to evolve in TPVR and SPVR, thus the incidence of mortality and severe outcomes is certainly an important metric of future research that needs further exploration as the technology changes.

The most recent systematic review and meta-analysis by *Çekirdekçi* et al [18] depicted that patients undergoing TPVR experienced a higher risk of IE compared to the SPVR group. This piece of evidence echoed with what we identified in our review. We performed further analysis by differentiating the risks into the early period and over the duration of follow-up; an increased risk of IE was noted during the follow-up time, but there was no significant difference at the early period. Such findings might be ascribed to the results from the studies by *Enezate* et al [25] and *Lluri* et al [26] that showed SPVR patients were at increased risk for IE, giving rise to a pooled estimate that showed a null effect. A previous study [18] suggested that younger patients were correlated with a higher incidence of IE. Three out of five studies incorporated into our meta-analysis predominantly recruited patients of younger age groups [39,42,50]. Notably, younger age, higher residual gradient and previous history of endocarditis are major risk factors for IE [54,55]. Besides, friction between valve and conduit, incomplete apposition of the valve against conduit and lack of valve re-endothelialization may contribute to IE after under-replaced TPVR [26,48]. It is likely that IE is caused by the environment where the valve is being placed than the valve itself [56].

Interestingly, while patients receiving TPVR were associated with a higher likelihood of IE compared with SPVR, the risk of re-intervention was similar among the groups. Indeed, patients who underwent re-intervention were not only caused by IE, but also other factors like prosthesis dysfunction, stent fracture, etc. [57] and some patients treated their symptoms at home with inappropriate oral antimicrobial therapy [6]. In consonance with the findings from previous systematic reviews and meta-analyses, we showed that the risk of re-intervention was not different between patients receiving either TPVR or SPVR in follow-up period. In comparison with a review by *Zhou* et al [16], we collated a larger number of studies in our analysis. Moreover, we applied different numbers of patients with re-intervention retrieved from a study *Coats* et al [42]. The primary studies that were included in our review were also different from the review by *Ribeiro*, et al [17]. The authors included 9 studies in their analysis and two of them were not included in ours, as one [53] was a letter to the editor and another study [24] assumed the rate of re-intervention based on a previous study. We also retrieved an additional study [42] that was not identified in Ribeiro et al review [17]. This could be the reason that led our pooled estimate of re-intervention (OR = 0.86) to be lower than *Zhou* et al study (OR = 2.19) [16] and higher than *Ribeiro* et al study (OR = 0.51) [17], although all detected no statistically significant differences.

To date, TPVR has increasingly been used in the context of patients with RV or RVOT dysfunctions who under-replace their valves. In the current systematic review and meta-analysis, we assessed the risk and benefit of utilizing TPVR based on significant pooled estimates of our results and demonstrated a greater risk of IE, without clinically meaningful survival benefits for the patients. It was noted that results across the clinical outcomes varied according to geographical region, publication year cut-off, and income status of country. To the best of our knowledge, no study has been performed to thoroughly evaluate the cost-effectiveness of TPVR as compared with SPVR, except for a cost analysis study by *Vergales* et al [58] that was published in 2013. The authors compared the costs of using TPVR and SPVR among 17 patients with a history of RVOT repair in the US for 10 years and depicted that those receiving TPVR had a substantial cost reduction of direct medical expenditures ($80,328 [±17,387] vs $126,406 [±38,772]) and total direct and indirect expenditures ($80,939 [±17,334] vs $129,519 [±39,021]). Although the effects of TPVR were evaluated in this study in tandem with risk-benefit assessment, the future challenge lies at the nexus of conducting a cost-effectiveness analysis of this and ethical, legal, economic, and sociocultural aspects have to be considered before the study implementation.

## Conclusions

Compared to SPVR, TPVR showed benefits in terms of mortality, however, it was associated with an increased risk of IE. Our findings are expected to provide healthcare professionals and policy makers with all presently available high-quality evidence on the clinical effects of TPVR to promote its integration and application in practice environments.

### Strengths and limitations

A major strength of our systematic review was that it employed a comprehensive search strategy to retrieve published literature that made direct comparisons between TPVR and SPVR. However, the pooled evidence was constrained by a sparsity of studies on the topic. As such, our subgroup analyses featured mainly on data from the United States and Europe, which implied that extrapolation of our results to other regions of the world, especially with varying healthcare policies, financing, and resources, could not reliably be executed. We did not search grey literature, thus there might be an omission of studies not published in indexed journals. Notwithstanding, we followed the PRISMA guidelines and reviewed a number of good-quality, most recent studies to lead to more transparent, complete, and accurate reporting that is aimed at improving evidence-based decision making in clinical practice. Fewer than half of the included studies had adjusted for important confounding variables, such as patient demographics, prior procedure, comorbid conditions, and primary diagnosis. We cannot completely rule out the possibility of residual confounding in the comparative effectiveness analysis (S10 Table). In fact, to perform heart valve replacement, patients were classified based on the structure of their heart and functioning, as well as individual backgrounds to ensure they received the appropriate surgical technique [17]. We did not have information to explore the surgical expertise in different hospital settings. There may be knowledge or experience gaps around TPVR and SPVR in studies from low-income or lower-middle income countries. Therefore, we were unable to fully reconcile heterogeneities of surgical expertise in our analysis, which may affect the pooled results for the clinical outcomes. Furthermore, we found a sparse number of studies (n = 2, 7.1%) from low-income and middle-income countries. Evidence is thus lacking at a global level to assist the formulation of a clearer roadmap for resource utilization and allocation to increase access to effective cardiovascular care and treatment-decision algorithms, particularly in developing and resource-poor countries where progress on interventional therapy for CHD has typically been stagnant. Caution should be exercised when interpreting the findings because the pooled estimates may be prone to bias due to residual confounding across the studies. The observational research design of all included studies is subject to confounding by indication or unmeasured confounding [59]. Randomized controlled trials are pivotal in advancing the evidence. Secondly, even though a total of 25 studies were included in our meta-analysis, some of the pooled estimates only had data from a few studies, limiting the generalizability of our findings across a wider array of patient populations and care settings. Furthermore, more than 90% of the included studies were conducted in high-income nations. Practical application of the research evidence should consider whether the healthcare settings match the characteristics of the facility and clinical environment of the individual studies. Third, our findings suggested that the duration of follow-up affected outcomes of the patients receiving valve replacement. Although we attempted to elucidate the impact of this in our subgroup analysis and the impact of different trade names of the valves given to the patients, novel evidence generation was not possible due to the scarcity of studies and data per individual patient.

Future research is therefore warranted to bridge the deficiencies and gaps so as to consolidate our present understanding of the follow-up duration for optimal patient monitoring and the various brand names of heart valves and their clinical applications. In addition, several non-English articles detected in our search results were excluded from this review, which reduced the number of studies available for this review and subjected it to language bias. Generally, studies with null or negative findings were more likely to be published in non-English-language journals [60] and the overall findings might potentially be skewed. Notwithstanding, we fervently opined that most of the high-quality studies had been published in English and were included in our review. This research is spotlighted by its value disparately: to provide healthcare professionals and policy makers with a clear insight into the data relevant to heart valve replacement, as well as the current evidence of the effects of TPVR compared with SPVR, allowing a holistic assessment of the net clinical advantage when using TPVR.

Driven by recent technical advancements and its suitability for a wide range of conditions and patient ages, TPVR has expanded its indications for managing PR, native or patched RVOTs, large conduits, and RVOT dysfunctions such as TOF [61,62]. It is noteworthy that the use of a transannular patch for the correction of TOF effectively relieves RVOT obstruction. However, it exposes patients to severe pulmonary valve regurgitation [63]. TPVR potentially offers favorable clinical outcomes with minimal invasiveness. With the advent of a new generation of self-expanding valve implants, including the Harmony Transcatheter Pulmonary Valve, the Alterra Adaptive Prestent system, the Venus P-Valve, and the Pulsta Valve, size limitations have been addressed. Such advancement enables the treatment of large, dilated native RVOTs [64] and broadens the use of TPVR.

PR following TOF repair can result in right ventricular dilation and failure, tricuspid regurgitation, reduced exercise performance, and arrhythmias, which can be prevented by a timely re-operation to insert a pulmonary valve [65]. Delaying surgical intervention leads to the occurrence of irreversible mechanoelectrical cardiomyopathy and poor prognosis [66]. Our review demonstrated no discernible differences between TPVR and SPVR in terms of post-operative PR or re-intervention. We conjecture that early TPVR intervention may be beneficial in preserving right ventricular function. Further research is therefore imperative to determine whether early intervention with TPVR or SPVR results in improved outcomes across diverse patient populations.

**Highlights**

- Our present systematic review distinguishes itself from existing research work by formal synthesis of quantitative evidence of early and late outcomes for mortality and other clinical endpoints, inclusion of studies published between 2019 and 2024, assessment of benefits and risks, as well as utilization of subgroup analyses to decipher the impact of TPVR and SPVR with respect to geographical region, World Bank income classification of countries, and time of publication.
- TPVR demonstrated a significant benefit on longer-term mortality. However, it was associated with an increased risk for infective endocarditis.
- A Monte Carlo simulation suggested TPVR would not gain benefits from survival outcome.
- Our findings provided an unparalleled insight into differences in the clinical impact of TPVR in different geographic areas, publication year, and country income level.

## Supporting information

S1 Table Search results. (PDF)

S2 Table A summary of the study outcomes: mortality. (PDF)

S3 Table A summary of the study outcomes: pulmonary regurgitation. (PDF)

S4 Table A summary of the study outcomes: infective endocarditis. (PDF)

S5 Table A summary of the study outcomes: re-intervention. (PDF)

S6 Table A summary of the study outcomes: the improvement in cardiac impairment according to NYHA functional classification. (PDF)

S7 Table A summary of the study outcomes: adverse events. (PDF)

S8 Table Estimation of incremental risk and benefit ratio between transcatheter pulmonary valve replacement and surgical pulmonary valve replacement. (PDF)

S9 Table Methodological quality assessment of the studies. (PDF)

S10 Table Domains of potential confounders observed in the studies. (PDF)

S1 Fig Forest plot of 30-day mortality based on region. OR, odds ratio.
(TIF)

S2 Fig Forest plot of the mortality during follow-up based on region. OR, odds ratio.
(TIF)

S3 Fig Forest plot of 30-day mortality based on year of publication. OR, odds ratio.
(TIF)

S4 Fig Forest plot of the mortality during follow-up based on year of publication. OR, odds ratio.
(TIF)

S5 Fig Forest plot of 30-day mortality based on income level. OR, odds ratio.
(TIF)

S6 Fig Forest plot of the mortality during follow-up based on income level. OR, odds ratio.
(TIF)

S7 Fig Forest plot of early pulmonary regurgitation based on region. OR, odds ratio.
(TIF)

S8 Fig Forest plot of pulmonary regurgitation during follow-up based on region. OR, odds ratio.
(TIF)

S9 Fig Forest plot of early pulmonary regurgitation based on year of publication. OR, odds ratio.
(TIF)

S10 Fig Forest plot of pulmonary regurgitation during follow-up based on year of publication. OR, odds ratio.
(TIF)

S11 Fig Forest plot of early pulmonary regurgitation based on income level. OR, odds ratio.
(TIF)

S12 Fig Forest plot of pulmonary regurgitation during follow-up based on income level. OR, odds ratio.
(TIF)

S13 Fig Forest plot of early infective endocarditis based on region. OR, odds ratio.
(TIF)

S14 Fig Forest plot of infective endocarditis during follow-up based on region. OR, odds ratio.
(TIF)

S15 Fig Forest plot of early infective endocarditis based on year of publication. OR, odds ratio.
(TIF)

S16 Fig Forest plot of infective endocarditis during follow-up based on year of publication. OR, odds ratio.
(TIF)

S17 Fig Forest plot of early infective endocarditis based on income level. OR, odds ratio.
(TIF)

S18 Fig Forest plot of infective endocarditis during follow-up based on income level. OR, odds ratio.
(TIF)

S19 Fig Forest plot of early re-intervention based on region. OR, odds ratio.
(TIF)

S20 Fig Forest plot of the re-intervention during follow-up based on region. OR, odds ratio.
(TIF)

S21 Fig Forest plot of early re-intervention based on year of publication. OR, odds ratio.
(TIF)

S22 Fig Forest plot of the re-intervention during follow-up based on year of publication. OR, odds ratio.
(TIF)

S23 Fig Forest plot of early re-intervention based on income level. OR, odds ratio.
(TIF)

S24 Fig Forest plot of the re-intervention during follow-up based on income level. OR, odds ratio.
(TIF)

S25 Fig Forest plot of the incremental risk; infective endocarditis. RD, risk difference.
(TIF)

S26 Fig Forest plot of the incremental benefits; survival. RD, risk difference.
(TIF)

S27 Fig Cost-effective plane demonstrating incremental infective endocarditis and survival at varying threshold levels. (TIF)
