# Supplementary material for: Comparative effects of transcatheter versus surgical pulmonary valve replacement: A systematic review and meta-analysis
Source: PLoS One. 2025 May 20;20(5):e0322041. doi: 10.1371/journal.pone.0322041 (PMC12091831; doi:10.1371/journal.pone.0322041)
Supplement: S1 Table — (PDF) [file pone.0322041.s001.pdf]

S1 Table. Search results.

PUBMED

No	Keywords	Found
1	pulmon* valve disease* OR right ventric* outflow tract dysfunction* OR right ventric* outflow tract obstruction OR tetralogy of Fallot OR truncus arteriosus OR transposition great arter* OR double outlet right ventric* OR pulmon* stenosis OR pulmon* regurgitation OR pulmon* insufficiency OR ventric* septal defect OR pulmon* atresia OR double chamber* right ventric*	137,179
2	percutaneous pulmon* valve OR transcatheter pulmon* valve	4,049
3	clinical outcome* OR clinical evaluation* OR clinical effect* OR periprocedural mortality OR mortality OR haemodynamic* OR hemodynamic* OR pulmo* stenosis OR pulmo* regurgitation OR exercise capacity OR MACE OR Major adverse cardiovascular event* OR serious adverse event* OR stent fracture OR infective endocarditis OR complication* OR pulmo* artery obstruction OR reintervention OR coronary artery compression OR valve embolization OR valve embolisation OR conduit rupture OR procedur* success OR stent fracture OR reintervention OR reoperation OR conduit rupture OR NYHA OR NYHA functional classification OR New York Heart Association	8,047,834
4	1 AND 2 AND 3	3,496

COCHRANE CENTRAL

No	Keywords	Found
1	pulmon* valve disease* OR right ventric* outflow tract dysfunction* OR right ventric* outflow tract obstruction OR tetralogy of Fallot OR truncus arteriosus OR transposition great arter* OR double outlet right ventric* OR pulmon* stenosis OR pulmon* regurgitation OR pulmon* insufficiency OR ventric* septal defect OR pulmon* atresia OR double chamber* right ventric*	6,563
2	percutaneous pulmon* valve OR transcatheter pulmon* valve	197
3	clinical outcome* OR clinical evaluation* OR clinical effect* OR periprocedural mortality OR mortality OR haemodynamic* OR hemodynamic* OR pulmo* stenosis OR pulmo* regurgitation OR exercise capacity OR MACE OR Major adverse cardiovascular event* OR serious adverse event* OR stent fracture OR infective endocarditis OR complication* OR pulmo* artery obstruction OR reintervention OR coronary artery compression OR valve embolization OR valve embolisation OR conduit rupture OR procedur* success OR stent fracture OR reintervention OR reoperation OR conduit rupture OR NYHA OR NYHA functional classification OR New York Heart Association	1,231,924
4	1 AND 2 AND 3	181

EMBASE

No	Keywords	Found
1	pulmon* valve disease* OR right ventric* outflow tract dysfunction* OR right ventric* outflow tract obstruction OR tetralogy of Fallot OR truncus arteriosus OR transposition great arter* OR double outlet right ventric* OR pulmon* stenosis OR pulmon* regurgitation OR pulmon* insufficiency OR ventric* septal defect OR pulmon* atresia OR double chamber* right ventric*	169,480
2	percutaneous pulmon* valve OR transcatheter pulmon* valve	11,619
3	clinical outcome* OR clinical evaluation* OR clinical effect* OR periprocedural mortality OR mortality OR haemodynamic* OR hemodynamic* OR pulmo* stenosis OR pulmo* regurgitation OR exercise capacity OR MACE OR Major adverse cardiovascular event* OR serious adverse event* OR stent fracture OR infective endocarditis OR complication* OR pulmo* artery obstruction OR reintervention OR coronary artery compression OR valve embolization OR valve	78,354

	embolisation OR conduit rupture OR procedur* success OR stent fracture OR reintervention OR reoperation OR conduit rupture OR NYHA OR NYHA functional classification OR New York Heart Association	
4	1 AND 2 AND 3	1,372

CINAHL COMPLETE

No	Keywords	Found
1	pulmon* valve disease* OR right ventric* outflow tract dysfunction* OR right ventric* outflow tract obstruction OR tetralogy of Fallot OR truncus arteriosus OR transposition great arter* OR double outlet right ventric* OR pulmon* stenosis OR pulmon* regurgitation OR pulmon* insufficiency OR ventric* septal defect OR pulmon* atresia OR double chamber* right ventric*	5,173
2	percutaneous pulmon* valve OR transcatheter pulmon* valve	175
3	clinical outcome* OR clinical evaluation* OR clinical effect* OR periprocedural mortality OR mortality OR haemodynamic* OR hemodynamic* OR pulmo* stenosis OR pulmo* regurgitation OR exercise capacity OR MACE OR Major adverse cardiovascular event* OR serious adverse event* OR stent fracture OR infective endocarditis OR complication* OR pulmo* artery obstruction OR reintervention OR coronary artery compression OR valve embolization OR valve embolisation OR conduit rupture OR procedur* success OR stent fracture OR reintervention OR reoperation OR conduit rupture OR NYHA OR NYHA functional classification OR New York Heart Association	1,171,690
4	1 AND 2 AND 3	51

WEB OF SCIENCE

No	Keywords	Found
1	pulmon* valve disease* OR right ventric* outflow tract dysfunction* OR right ventric* outflow tract obstruction OR tetralogy of Fallot OR truncus arteriosus OR transposition great arter* OR double outlet right ventric* OR pulmon* stenosis OR pulmon* regurgitation OR pulmon* insufficiency OR ventric* septal defect OR pulmon* atresia OR double chamber* right ventric*	61,525
2	percutaneous pulmon* valve OR transcatheter pulmon* valve	3,543
3	clinical OR mortality OR haemodynamic* OR hemodynamic* OR pulmo* stenosis OR pulmo* regurgitation OR exercise capacity OR MACE OR Major adverse cardiovascular event* OR serious adverse event* OR stent fracture OR infective endocarditis OR complication* OR pulmo* artery obstruction OR reintervention OR coronary artery compression OR valve embolization OR valve embolisation OR conduit rupture OR procedur* success OR stent fracture OR reintervention OR reoperation OR conduit rupture OR NYHA OR New York Heart Association	9,483,103
4	1 AND 2 AND 3	2,668

Note: A detailed inventory of studies identified through electronic literature searches, registry databases, and manual hand-searching, including documented reasons for exclusion, is presented in the accompanying table accessible via the following Google link: https://docs.google.com/spreadsheets/d/1-CkIxGuNO6jy4qhY9Af0eik7HRPO-0Ow/edit?usp=sharing&ouid=116504548655118233101&rtpof=true&sd=true
