# Supplementary material for: Comparative effects of transcatheter versus surgical pulmonary valve replacement: A systematic review and meta-analysis
Source: PLoS One. 2025 May 20;20(5):e0322041. doi: 10.1371/journal.pone.0322041 (PMC12091831; doi:10.1371/journal.pone.0322041)
Supplement: S2 Table — (PDF) [file pone.0322041.s002.pdf]

S2 Table. A summary of the study outcomes: mortality.

First author (y)	Trade name comparisons		Sample size (TPVR vs SPVR)	Follow-up duration (months)	Mortality (n)	
	TPVR	SPVR			TVPR	SPVR
30-day mortality						
Alassas (2018) [48]	Melody	NR	47 vs 41	TPVR = 56.0 ± 24.0<br>SPVR = 89.0 ± 46.0	0	0
Caughron (2018) [23]	1) Melody<br>2) SAPIEN	1) Contegra<br>2) Homograft<br>3) Mosaic/Hancock<br>4) Perimount Magna<br>5) Trifecta	36 vs 30	25.9 (IQR: 12.25, 46.45)	1	0
Coats (2005) [42]	NR	NR	35 vs 94	TPVR = 4.0 (range: 0.1, 59.5)<br>SPVR = 10.0 (range: 0.1, 14.3)	0	1
Durongpisitkul (2022) [50]	1) Melody<br>2) Pulsta<br>3) SAPIEN<br>4) Venus P-valve	1) Contegra<br>2) Freestyle bioprosthesis<br>3) Homograft<br>4) Perimount Magna	72 vs 143	24	0	8
Hribernik (2022) [43]	1) Melody<br>2) SAPIEN	NR	120 vs 365	TPVR = 17 (range: 0, 116)<br>SPVR = 47 (range: 0, 243)	0	4
Lluri (2018) [26]	1) Melody<br>2) SAPIEN	NR	208 vs 134	TPVR = 26.4 (IQR: 1.0, 3.1)<br>SPVR = 33.6 (IQR: 0.9, 4.0)	0	1
Skoglund (2017) [49]	NR	NR	50 vs 762	190.8 (95% CI: 181.2, 200.4)	0	3
Steinberg (2017) [32]	Melody	NR	78 vs 145	NR	0	4
Wadia (2018) [34]	NR	NR	172 vs 130	TPVR = 15.6 (IQR: 2.4, 33.6)<br>SPVR = 12.0 (IQR: 1.2, 34.8)	0	2
Mortality over the duration of follow-up						
Alassas (2018) [48]	Melody	NR	47 vs 41	TPVR = 56.0 ± 24.0<br>SPVR = 89.0 ± 46.0	2	1
Andressen (2018) [47]	1) Melody<br>2) SAPIEN	1) Contegra<br>2) Homograft<br>3) Perimount Magna	20 vs 14	12	0	0
Caughron (2018) [23]	1) Melody<br>2) SAPIEN	1) Contegra<br>2) Homograft<br>3) Mosaic/Hancock	36 vs 30	25.9 (IQR: 12.25, 46.45)	1	0

First author (y)	Trade name comparisons		Sample size (TPVR vs SPVR)	Follow-up duration (months)	Mortality (n)	
	TPVR	SPVR			TVPR	SPVR
		4) Perimount Magna<br>5) Trifecta				
Daily (2018) [24]	Melody	NR	191 vs 382	NR	1	3
Durongpisitkul (2022) [50]	1) Melody<br>2) Pulsta<br>3) SAPIEN<br>4) Venus P-valve	1) Contegra<br>2) Freestyle bioprosthesis<br>3) Homograft<br>4) Perimount Magna	72 vs 143	24	2	10
Enezate (2019) [25]	NR	NR	176 vs 799	NR	0	11
Georgiev (2020) [38]	Melody	1) Contegra<br>2) Hancock<br>3) Homograft<br>4) Others	241 vs 211	TPVR = 57.6 (range: 2.4, 139.2)<br>SPVR = 76.8 (range: 2.4, 151.2)	7	11
Hribernik (2022) [43]	1) Melody<br>2) SAPIEN	NR	120 vs 365	TPVR = 17 (range: 0, 116)<br>SPVR = 47 (range: 0, 243)	6	16
Malekzadeh-Milani (2014) [45]	Melody	NR	93 vs 195	TPVR = 23.8 (95% CI: 17.5, 32.5)<br>SPVR = 24.1 (95% CI: 19.9, 29.9)	4	11
Megaly (2021) [27]	1) Melody<br>2) SAPIEN	NR	1140 vs 4305	NR	10	69
O'byrne (2015) [28]	NR	NR	30 vs 769	NR	0	7
O'Byrne (2016) [29]	NR	NR	292 vs 1816	NR	0	36
Ou-Yang (2020) [44]	Venus P-valve	Homograft	35 vs 30	TPVR = 36 (IQR: 36, 48)<br>SPVR = 36 (IQR: 33, 48)	0	0
Sharma (2018) [30]	Melody	NR	124 vs 100	TPVR = 18.7 ± 17.0<br>SPVR = 31.6 ± 22.0	0	0
Sosnowski (2016) [31]	Melody	NR	8 vs 13	TPVR = 3.4 ± 4.58<br>SPVR = 13.6 ±11.98	0	0
Van Dijck (2014) [33]	Melody	1) Contegra<br>2) Homograft	107 vs 631	TPVR<br>- Melody = 24.0<br>SPVR<br>- Contegra = 78.0<br>- Homograft = 105.6	0	16

IQR, interquartile range*; NR*, no report; *SPVR*, surgical pulmonary valve replacement; *TPVR*, transcatheter pulmonary valve replacement
