# Supplementary material for: Comparative effects of transcatheter versus surgical pulmonary valve replacement: A systematic review and meta-analysis
Source: PLoS One. 2025 May 20;20(5):e0322041. doi: 10.1371/journal.pone.0322041 (PMC12091831; doi:10.1371/journal.pone.0322041)
Supplement: S3 Table — (PDF) [file pone.0322041.s003.pdf]

**S3 Table.** A summary of the study outcomes: pulmonary regurgitation.

| **First author (y)** | **Trade name** | | **Sample size (TPVR vs SPVR)** | **Follow-up duration (months)** | **Pulmonary regurgitation (n)** | |
|---|---|---|---|---|---|---|
| | **TPVR** | **SPVR** | | | **TPVR** | **SPVR** |
| ***Early pulmonary regurgitation*** | | | | | | |
| Caughron (2018) [23] | 1) Melody<br>2) SAPIEN | 1) Contegra<br>2) Homograft<br>3) Mosaic/Hancock<br>4) Perimount Magna<br>5) Trifecta | 36 vs 30 | 25.9 (IQR: 12.25, 46.45) | ≥ Moderate = 0 | ≥ Moderate = 0 |
| Durongpisitkul (2022) [50] | 1) Melody<br>2) Pulsta<br>3) SAPIEN<br>4) Venus P-valve | 1) Contegra<br>2) Freestyle bioprosthesis<br>3) Homograft<br>4) Perimount Magna | 72 vs 143 | 24 | - None = 62<br>- Mild = 5<br>- Moderate = 3<br>- Severe = 0 | - None = 65<br>- Mild = 30<br>- Moderate = 9<br>- Severe = 1 |
| Hribernik (2022) [43] | 1) Melody<br>2) SAPIEN | NR | 120 vs 365 | TPVR = 17 (range: 0, 116)<br>SPVR = 47 (range: 0, 243) | - None = 84<br>- Mild = 34<br>- Moderate = 2<br>- Severe = 0 | - None = 134<br>- Mild = 216<br>- Moderate = 8<br>- Severe = 7 |
| ***Pulmonary regurgitation over the duration of follow-up*** | | | | | | |
| Alassas (2018) [48] | Melody | NR | 47 vs 41 | TPVR = 56.0 ± 24.0<br>SPVR = 89.0 ± 46.0 | ≥ Moderate = 0 | ≥ Moderate = 13 |
| Caughron (2018) [23] | 1) Melody<br>2) SAPIEN | 1) Contegra<br>2) Homograft<br>3) Mosaic/Hancock<br>4) Perimount Magna<br>5) Trifecta | 36 vs 30 | 25.9 (IQR: 12.25, 46.45) | ≥ Moderate = 2 | ≥ Moderate = 3 |
| Durongpisitkul (2022) [50] | 1) Melody<br>2) Pulsta<br>3) SAPIEN<br>4) Venus P-valve | 1) Contegra<br>2) Freestyle bioprosthesis<br>3) Homograft<br>4) Perimount Magna | 72 vs 143 | 24 | - None = 60<br>- Mild = 10<br>- Moderate = 1<br>- Severe = 1 | - None = 54<br>- Mild = 48<br>- Moderate = 4<br>- Severe = 5 |
| Egbe (2024) [37] | 1) Melody<br>2) SAPIEN | NR | 51 vs 113 | 36 | ≥ Moderate = 4 | ≥ Moderate = 3 |
| Gröning (2024) [41] | 1) Melody<br>2) SAPIEN | 1) Homograft<br>2) Contegra<br>3) Perimount/Magna<br>4) Magna Ease<br>5) Hancock<br>6) Freestyle | 14 vs 148 | 124.8 (IQR: 43.2, 198) | None = 3 | None = 16 |

| First author (y) | Trade name | | Sample size (TPVR vs SPVR) | Follow-up duration (months) | Pulmonary regurgitation (n) | |
|---|---|---|---|---|---|---|
| | TPVR | SPVR | | | TPVR | SPVR |
| Hribernik (2022) [43] | 1) Melody<br>2) SAPIEN | NR | 120 vs 365 | TPVR = 17 (range: 0, 116)<br>SPVR = 47 (range: 0, 243) | - None = 81<br>- Mild = 37<br>- Moderate = 2<br>- Severe = 0 | - None = 73<br>- Mild = 204<br>- Moderate = 74<br>- Severe = 14 |
| Lluri (2018) [26] | 1) Melody<br>2) SAPIEN | NR | 208 vs 134 | TPVR = 26.4 (IQR: 1.0, 3.1)<br>SPVR = 33.6 (IQR: 0.9, 4.0) | Severe = 0 | Severe = 1 |
| Sosnowski (2016) [31] | Melody | NR | 8 vs 13 | TPVR = 3.4 ± 4.58<br>SPVR = 13.6 ±11.98 | - None = 5<br>- Mild = 3<br>- Moderate = 0<br>- Severe = 0 | - None = 3<br>- Mild = 8<br>- Moderate = 0<br>- Severe = 0 |

*IQR*, interquartile range; *NR*, no report; *SPVR*, surgical pulmonary valve replacement; *TPVR*, transcatheter pulmonary valve replacement
