# Supplementary material for: Comparative effects of transcatheter versus surgical pulmonary valve replacement: A systematic review and meta-analysis
Source: PLoS One. 2025 May 20;20(5):e0322041. doi: 10.1371/journal.pone.0322041 (PMC12091831; doi:10.1371/journal.pone.0322041)
Supplement: S4 Table — (PDF) [file pone.0322041.s004.pdf]

S4 Table. A summary of the study outcomes: infective endocarditis.

First author (y)	Trade name		Sample size (TPVR vs SPVR)	Follow-up duration (months)	Infective endocarditis (n)	
	TPVR	SPVR			TPVR	SPVR
Early infective endocarditis						
Caughron (2018) [23]	1) Melody<br>2) SAPIEN	1) Contegra<br>2) Homograft<br>3) Mosaic/Hancock<br>4) Perimount Magna<br>5) Trifecta	36 vs 30	25.9 (IQR: 12.25, 46.45)	0	0
Coats (2005) [42]	NR	NR	35 vs 94	TPVR = 4.0 (range: 0.1, 59.5)<br>SPVR = 10.0 (range: 0.1, 14.3)	1	0
Durongpisitkul (2022) [50]	1) Melody<br>2) Pulsta<br>3) SAPIEN<br>4) Venus P-valve	1) Contegra<br>2) Freestyle bioprosthesis<br>3) Homograft<br>4) Perimount Magna	72 vs 143	24	1	1
Enezate (2019) [25]	NR	NR	176 vs 799	NR	3	25
Haas (2018) [39]	1) Melody<br>2) SAPIEN	1) Contegra<br>2) Hancock<br>3) Homograft	80 vs 166	TPVR<br>- Melody = 51.6<br>- SAPIEN = 28.8<br>SPVR<br>- Contegra = 37.2<br>- Hancock = 2.4<br>- Homograft = 20.0	1	0
Lluri (2018) [26]	1) Melody<br>2) SAPIEN	NR	208 vs 134	TPVR = 26.4 (IQR: 1.0, 3.1)<br>SPVR = 33.6 (IQR: 0.9, 4.0)	0	1
Infective endocarditis over the duration of follow-up						
Alassas (2018) [48]	Melody	NR	47 vs 41	TPVR = 56.0 ± 24.0<br>SPVR = 89.0 ± 46.0	6	0
Andresen (2018) [47]	1) Melody<br>2) SAPIEN	1) Contegra<br>2) Homograft<br>3) Perimount Magna	20 vs 14	12	0	0
Caughron (2018) [23]	1) Melody<br>2) SAPIEN	1) Contegra<br>2) Homograft<br>3) Mosaic/Hancock<br>4) Perimount Magna<br>5) Trifecta	36 vs 30	25.9 (IQR: 12.25, 46.45)	0	0

First author (y)	Trade name		Sample size (TPVR vs SPVR)	Follow-up duration (months)	Infective endocarditis (n)	
	TPVR	SPVR			TPVR	SPVR
Egbe (2024) [37]	1) Melody<br>2) SAPIEN	NR	64 vs 128	36	8	7
Georgiev (2020) [38]	Melody	1) Contegra<br>2) Hancock<br>3) Homograft<br>4) Others	241 vs 211	TPVR = 57.6 (range: 2.4, 139.2)<br>SPVR = 76.8 (range: 2.4, 151.2)	18	6
Gröning (2019) [40]	Melody	1) Contegra<br>2) Homograft	64 vs 368	TPVR<br>- Melody = 46.8 (IQR: 1.0, 6.8)<br>SPVR<br>- Contegra = 72.0 (IQR: 38.4, 98.4)<br>- Homograph = 8.3 (IQR: 3.6, 13.1)	7	16
Gröning (2024) [41]	1) Melody<br>2) SAPIEN	1) Homograft<br>2) Contegra<br>3) Perimount/Magna<br>4) Magna Ease<br>5) Hancock<br>6) Freestyle	14 vs 148	124.8 (IQR: 43.2, 198)	6	11
Haas (2018) [39]	1) Melody<br>2) SAPIEN	1) Contegra<br>2) Hancock<br>3) Homograft	80 vs 166	TPVR<br>- Melody = 51.6<br>- SAPIEN = 28.8<br>SPVR<br>- Contegra = 37.2<br>- Hancock = 2.4<br>- Homograft = 20.0	6	5
Hribernik (2022) [43]	1) Melody<br>2) SAPIEN	NR	120 vs 365	TPVR = 17 (range: 0, 116)<br>SPVR = 47 (range: 0, 243)	4	4
Lluri (2018) [26]	1) Melody<br>2) SAPIEN	NR	208 vs 134	TPVR = 26.4 (IQR: 1.0, 3.1)<br>SPVR = 33.6 (IQR: 0.9, 4.0)	7	2
Malekzadeh-Milani (2014) [45]	Melody	NR	93 vs 195	TPVR = 23.8 (95% CI: 17.5, 32.5)<br>SPVR = 24.1 (95% CI: 19.9, 29.9)	8	5
O'Donnell (2017) [46]	Melody	NR	25 vs 178	35.5 (range: 3, 67)	4	4
Ou-Yang (2020) [44]	Venus P-valve	Homograft	35 vs 30	TPVR = 36 (IQR: 36, 48)<br>SPVR = 36 (IQR: 33, 48)	1	0
Sharma (2018) [30]	Melody	NR	124 vs 100	TPVR = 18.7 ± 17.0<br>SPVR = 31.6 ± 22.0	6	0

First author (y)	Trade name		Sample size (TPVR vs SPVR)	Follow-up duration (months)	Infective endocarditis (n)	
	TPVR	SPVR			TPVR	SPVR
Van Dijck (2014) [33]	Melody	1) Contegra<br>2) Homograft	107 vs 631	TPVR<br>- Melody = 24.0<br>SPVR<br>- Contegra = 78.0<br>- Homograft = 105.6	8	25

IQR, interquartile range; *NR*, no report; *SPVR*, surgical pulmonary valve replacement; *TPVR*, transcatheter pulmonary valve replacement
