# Supplementary material for: Comparative effects of transcatheter versus surgical pulmonary valve replacement: A systematic review and meta-analysis
Source: PLoS One. 2025 May 20;20(5):e0322041. doi: 10.1371/journal.pone.0322041 (PMC12091831; doi:10.1371/journal.pone.0322041)
Supplement: S5 Table — (PDF) [file pone.0322041.s005.pdf]

S5 Table. A summary of the study outcomes: re-intervention.

First author (y)	Trade name		Sample size (TPVR vs SPVR)	Follow-up duration (months)	Re-intervention (n)	
	TPVR	SPVR			TPVR	SPVR
Early re-intervention						
Caughron (2018) [23]	1) Melody<br>2) SAPIEN	1) Contegra<br>2) Homograft<br>3) Mosaic/Hancock<br>4) Perimount Magna<br>5) Trifecta	36 vs 30	25.9 (IQR: 12.25, 46.45)	0	2
Durongpisitkul (2022) [50]	1) Melody<br>2) Pulsta<br>3) SAPIEN<br>4) Venus P-valve	1) Contegra<br>2) Freestyle bioprosthesis<br>3) Homograft<br>4) Perimount Magna	72 vs 143	24	5	13
O'Byrne (2016) [29]	NR	NR	292 vs 1816	NR	3	3
Re-intervention over the duration of follow-up						
Alassas (2018) [48]	Melody	NR	47 vs 41	TPVR = 56.0 ± 24.0<br>SPVR = 89.0 ± 46.0	10	5
Andressen (2018) [47]	1) Melody<br>2) SAPIEN	1) Contegra<br>2) Homograft<br>3) Perimount Magna	20 vs 14	12	0	1
Caughron (2018) [23]	1) Melody<br>2) SAPIEN	1) Contegra<br>2) Homograft<br>3) Mosaic/Hancock<br>4) Perimount Magna<br>5) Trifecta	36 vs 30	25.9 (IQR: 12.25, 46.45)	0	3
Coats (2005) [42]	NR	NR	35 vs 94	TPVR = 4.0 (range: 0.1, 59.5)<br>SPVR = 10.0 (range: 0.1, 14.3)	4	1
Durongpisitkul (2022) [50]	1) Melody<br>2) Pulsta<br>3) SAPIEN<br>4) Venus P-valve	1) Contegra<br>2) Freestyle bioprosthesis<br>3) Homograft<br>4) Perimount Magna	72 vs 143	24	7	14
Egbe (2024) [37]	1) Melody<br>2) SAPIEN	NR	64 vs 128	108	5	8
Georgiev (2020) [38]	Melody	1) Contegra<br>2) Hancock<br>3) Homograft<br>4) Others	241 vs 211	TPVR = 57.6 (range: 2.4, 139.2)<br>SPVR = 76.8 (range: 2.4, 151.2)	18	24

First author (y)	Trade name		Sample size (TPVR vs SPVR)	Follow-up duration (months)	Re-intervention (n)	
	TPVR	SPVR			TPVR	SPVR
Hribernik (2022) [43]	1) Melody<br>2) SAPIEN	NR	120 vs 365	TPVR = 17 (range: 0, 116)<br>SPVR = 47 (range: 0, 243)	15	54
Li (2017) [35]	Melody	NR	32 vs 30	TPVR = 24.8 (range: 7.1, 46.6)<br>SPVR = 23.2 (range: 7.4, 49.4)	0	3
Sharma (2018) [30]	Melody	NR	124 vs 100	TPVR = 18.7 ± 17.0<br>SPVR = 31.6 ± 22.0	4	5
Van Dick (2014) [33]	Melody	1) Contegra<br>2) Homograft	107 vs 631	TPVR<br>- Melody = 24.0<br>SPVR<br>- Contegra = 78.0<br>- Homograft = 105.6	0	16

IQR, interquartile range*; NR*, no report; *SPVR*, surgical pulmonary valve replacement; *TPVR*, transcatheter pulmonary valve replacement
