# Supplementary material for: Comparative effects of transcatheter versus surgical pulmonary valve replacement: A systematic review and meta-analysis
Source: PLoS One. 2025 May 20;20(5):e0322041. doi: 10.1371/journal.pone.0322041 (PMC12091831; doi:10.1371/journal.pone.0322041)
Supplement: S6 Table — (PDF) [file pone.0322041.s006.pdf]

S6 Table. A summary of the study outcomes: the improvement in cardiac impairment according to NYHA functional classification.

First author (y)	Trade name		Sample size (TPVR vs SPVR)	Follow-up duration (months)	NYHA functional classification (class 1–4) (n)			
					TPVR		SPVR	
	TPVR	SPVR			Baseline	Final	Baseline	Final
Early improvement of cardiac impairment according to NYHA functional classification								
Caughron (2018) [23]	1) Melody<br>2) SAPIEN	1) Contegra<br>2) Homograft<br>3) Mosaic/Hancock<br>4) Perimount Magna<br>5) Trifecta	36 vs 30	25.9 (IQR: 12.25, 46.45)	Class 3-4 = 27	Class 3-4 = 1	Class 3-4 = 10	Class 3-4 = 1
Hribernik (2022) [43]	1) Melody<br>2) SAPIEN	NR	120 vs 365	TPVR = 17 (range: 0, 116)<br>SPVR = 47 (range: 0, 243)	Class 1 = 57	Class 1 = 168	Class 1 = 41	Class 1 = 118
Lluri (2018) [26]	1) Melody<br>2) SAPIEN	NR	208 vs 134	TPVR = 26.4 (IQR: 1.0, 3.1)<br>SPVR = 33.6 (IQR: 0.9, 4.0)	Class 1 = 48<br>Class 2 = 40<br>Class 3 = 26<br>Class 4 = 6	Class 1 = 91<br>Class 2 = 27<br>Class 3 = 1<br>Class 4 = 1	Class 1 = 194<br>Class 2 = 131<br>Class 3 = 34<br>Class 4 = 6	Class 1 = 307<br>Class 2 = 52<br>Class 3 = 3<br>Class 4 = 3
The improvement in cardiac impairment according to NYHA functional classification over the duration of follow-up								
Caughron (2018) [23]	1) Melody<br>2) SAPIEN	1) Contegra<br>2) Homograft<br>3) Mosaic/Hancock<br>4) Perimount Magna<br>5) Trifecta	36 vs 30	25.9 (IQR: 12.25, 46.45)	Class 3-4 = 27	Class 3-4 = 0	Class 3-4 = 10	Class 3-4 = 0
Hribernik (2022) [43]	1) Melody<br>2) SAPIEN	NR	120 vs 365	TPVR = 17 (range: 0, 116)<br>SPVR = 47 (range: 0, 243)	Class 1 = 57	Class 1 = 168	Class 1 = 41	Class 1 = 118
Lluri (2018) [26]	1) Melody<br>2) SAPIEN	NR	208 vs 134	TPVR = 26.4 (IQR: 1.0, 3.1)<br>SPVR = 33.6 (IQR: 0.9, 4.0)	Class 1 = 1<br>Class 2 = 27<br>Class 3 = 7	Class 3-4 = 33<br>Class 3-4 = 2<br>Class 3-4 = 0	Class 3-4 = 4<br>Class 3-4 = 24<br>Class 3-4 = 2	Class 3-4 = 28<br>Class 3-4 = 2<br>Class 3-4 = 0
Ou-Yang (2020) [44]	Venus P-valve	Homograft	35 vs 30	TPVR = 36 (IQR: 36, 48)<br>SPVR = 36 (IQR: 33, 48)	Class 1 = 48<br>Class 1 = 40<br>Class 1 = 26<br>Class 1 = 6	Class 1 = 90<br>Class 1 = 23<br>Class 1 = 6<br>Class 1 = 1	Class 1 = 194<br>Class 1 = 131<br>Class 1 = 34<br>Class 1 = 6	Class 1 = 317<br>Class 1 = 31<br>Class 1 = 9<br>Class 1 = 8

IQR, interquartile range*; NR*, no report; *NYHA*, New York Heart Association*; SPVR*, surgical pulmonary valve replacement; *TPVR*, transcatheter pulmonary valve replacement
