# Supplementary material for: Comparative effects of transcatheter versus surgical pulmonary valve replacement: A systematic review and meta-analysis
Source: PLoS One. 2025 May 20;20(5):e0322041. doi: 10.1371/journal.pone.0322041 (PMC12091831; doi:10.1371/journal.pone.0322041)
Supplement: S7 Table — (PDF) [file pone.0322041.s007.pdf]

S7 Table. A summary of the study outcomes: adverse events.

First author (y)	Reported adverse events	TPVR (%)	SPVR (%)
Alassas (2018) [48]	Failure of transcatheter valve	2.1	0.0
	Femoral artery pseudoaneurysm	2.1	0.0
	Femoral AV fistula	2.1	0.0
	Stent fracture of prosthetic valve	2.1	0.0
	Sternal wound infection	0.0	12.2
Andresen (2018) [47]	Bleeding requiring reoperation and blood transfusion	0.0	7.1
	Pacemaker implantation	0.0	7.1
Bou Chaaya (2023) [36]	Ventricular tachycardia	7.4	10.3
Caughron (2018) [23]	Major vascular complications	0.0	3.3
	Major bleeding	0.0	13.3
	Major stroke	2.8	0.0
	Minor bleeding	2.8	23.3
	New atrial fibrillation/flutter	2.8	13.3
	Pacemaker implantation	2.8	6.7
Coats (2005) [42]	Acute psychosis	0.0	1.1
	Acute renal failure requiring hemodialysis	0.0	1.1
	Arrhythmia	0.0	8.5
	Chest infection	0.0	9.6
	Complete heart block requiring permanent pacemaker implantation	0.0	1.1
	Early in-stent stenosis	2.9	0.0
	Endocarditis	2.9	0.0
	Late RVOT restenosis	17.1	0.0
	Neurological events	0.0	2.1
	Pericarditis	0.0	2.1
	Pericardial effusion	0.0	4.3
	Pleural effusion	0.0	16.0
	Pneumothorax	0.0	5.3
	Prolonged temperature associated with blood culture yielding Candida albicans	2.9	0.0
	Pseudoaneurysm requiring surgical intervention	0.0	1.1
	Stent fracture	2.9	0.0
	Sudden hemodynamic collapse requiring resternotomy	0.0	1.1
	Systematic inflammatory response syndrome	0.0	1.1
	Tracheostomy and prolonged ventilation support	0.0	2.1
	Vocal cord palsy	0.0	2.1
Durongpisitkul (2022) [50]	All major complications requiring cardiopulmonary resuscitation, prolong ICU or hospital stay	11.1	32.9
Egbe (2024) [37]	Prosthetic valve dysfunction	17.2	9.4
	Surgical explantation of infected bioprosthesis	4.7	3.1
	Transcatheter debulking of vegetation	3.1	0.0

First author (y)	Reported adverse events	TPVR (%)	SPVR (%)
Enezate (2019) [25]	Bleeding	4.6	26.4
	Mechanical complications of heart valve prosthesis (valve dislodgement, embolization, and significant stent fracture)	1.7	2.0
	Vascular complications (conduit rupture, access site complications, and pulmonary artery injury)	2.3	2.0
Gröning (2024) [41]	Pulmonary stenosis	28.6	64.9
	Combined stenosis/regurgitation	7.1	12.8
Haas (2018) [39]	Cardiac complications	42.4	29.1
	Hemorrhage/hematoma	4.7	6.3
	Infection/fever	1.0	5.0
	Respiratory complications	4.2	23.3
	Other (not specified)	3.7	11.5
Hribernik (2022) [43]	Compartment syndrome in left leg	0.0	0.3
	Conduit rupture after stent implantation	0.8	0.0
	Coronary artery injury requiring intraoperative repair	0.0	0.3
	Displaced atrial pacing wire	0.8	0.0
	Embolization of catheter introduced device	0.8	0.0
	Iatrogenic ventricular injury requiring pericardial patch	0.0	0.3
	Mediastinal hematoma requiring sternotomy	0.0	0.3
	Mediastinitis/sternal infection	0.0	0.8
	Pericardial effusion requiring pericardiocentesis	0.0	0.5
	Perinephric hematoma	0.8	0.0
	Postoperative bradycardia requiring permanent pacemaker	0.0	0.3
	Postoperative stroke	0.0	0.5
	Postoperative hypotension requiring resuscitation and chest reopening	0.0	0.3
	Postprocedural death within 30 days	0.0	1.1
	Pulmonary edema	0.8	0.0
	Right brachial nerve palsy	0.8	0.0
	RVOT obstruction due to rotation of pulmonary valve requiring removal of the valve	0.0	0.3
	Sternal dehiscence	0.0	0.3
	Wire injury to right lower lobe requiring coil occlusion	0.8	0.0
Megaly (2021) [27]	Respiratory complications	3.9	16.6
	Vascular complications requiring surgery	0.4	0.2
	Ventricular arrhythmia	12.3	11.1
O'Donnell (2017) [46]	RVOT perforation	8.0	0.0
	Supraventricular tachycardia	8.0	0.0
	Ventricular tachycardia	4.0	0.0
Ou-Yang (2020) [44]	Atrial flutter	2.9	3.3
	Pericardial effusion	0.0	6.7
Sharma (2018) [30]	Acute right coronary artery injury during repeat sternotomy	0.0	1.0
	Embolization of the pre-stent	1.6	0.0

First author (y)	Reported adverse events	TPVR (%)	SPVR (%)
	High-degree heart block requiring pacemaker implantation	0.0	1.0
	Perforation of the preexisting RVPA conduit	1.6	0.0
	Recurrent pneumothorax	0.0	1.0
Steinberg (2017) [32]	Bleeding or infection requiring mediastinal exploration	0.0	1.4
	Cardiopulmonary arrest with crushed valve from chest compressions	1.3	0.0
	Cardiopulmonary arrest with residual neurological deficits	0.0	1.4
	Conduit rupture with procedure cessation prior to valve implantation	1.3	0.0
	Death within 30 days	0.0	2.8
	Heart block requiring permanent pacemaker implantation	0.0	1.4
	Hemidiaphragmatic paralysis	0.0	0.7
	Hemothorax, pneumothorax, or pleural effusion requiring chest tube placement	1.3	3.4
Wadia (2018) [34]	Non-intraoperative postprocedural events that impacted patient comfort, functional status, or length of hospitalization	12.8	32.3
