# Supplementary material for: Comparative effects of transcatheter versus surgical pulmonary valve replacement: A systematic review and meta-analysis
Source: PLoS One. 2025 May 20;20(5):e0322041. doi: 10.1371/journal.pone.0322041 (PMC12091831; doi:10.1371/journal.pone.0322041)
Supplement: S8 Table — (PDF) [file pone.0322041.s008.pdf]

S8 Table. Estimation of incremental risk and benefit ratio between transcatheter pulmonary valve replacement and surgical pulmonary valve replacement.

	**TPVR**		**SPVR**		**Incremental (95% CI)**	**Incremental risk and benefit ratio (95% CrI)**
	**N**	**Total**	**N**	**Total**		**Survival**
Risk						
Infective endocarditis	93	1350	89	2882	0.038 (0.023, 0.053)	4.222 (2.048, 12.318)
Benefits						
Death	33	2410	148	7259	0.009 (0.004, 0.013)	NA

CI, confidence interval; CrI, credible interval; NA, not applicable; *TPVR*, transcatheter pulmonary valve replacement; *SPVR*, surgical pulmonary valve replacement
