# Supplementary material for: Comparative effects of transcatheter versus surgical pulmonary valve replacement: A systematic review and meta-analysis
Source: PLoS One. 2025 May 20;20(5):e0322041. doi: 10.1371/journal.pone.0322041 (PMC12091831; doi:10.1371/journal.pone.0322041)
Supplement: S9 Table — (PDF) [file pone.0322041.s009.pdf]

S9 Table. Methodological quality assessment of the studies.

First author (y)	Selection				Comparability	Outcomes/exposures			Results
	1	2	3	4	1	1	2	3	
Alassas (2018) [48]	*	*	*	*	**	*	*	*	Good
Andresen (2018) [47]	*	*	-	-	**	*	*	-	Fair
Bou Chaaya (2023) [36]	*	*	*	*	*	*	*	-	Good
Caughron (2018) [23]	*	*	*	*	*	*	*	-	Good
Coats (2005) [42]	*	*	*	-	-	*	-	*	Poor
Daily (2018) [24]	*	*	*	*	*	*	-	*	Good
Durongpisitkul (2022) [50]	*	*	*	-	-	*	*	*	Poor
Egbe (2024) [37]	*	*	*	*	*	*	*	*	Good
Enezate (2019) [25]	*	*	*	-	**	*	-	-	Poor
Georgiev (2020) [38]	*	*	*	*	**	*	*	*	Good
Gröning (2019) [40]	*	*	*	-	-	*	*	*	Poor
Gröning (2024) [41]	*	*	*	*	*	*	*	*	Good
Haas (2018) [39]	*	*	*	-	-	*	-	*	Poor
Hribernik (2022) [43]	*	*	*	-	-	*	*	*	Poor
Li (2017) [35]	*	*	*	*	*	*	*	-	Good
Lluri (2018) [26]	*	*	*	*	**	*	-	-	Poor
Malekzadeh-Milani (2014) [45]	*	*	*	*	-	*	*	*	Poor
Megaly (2021) [27]	*	*	*	*	-	*	-	-	Poor
O'Byrne (2015) [28]	*	*	*	*	-	*	-	*	Poor
O'Byrne (2016) [29]	*	*	*	*	*	*	*	-	Good
O'Donnell (2017) [46]	*	*	*	-	-	*	*	*	Poor
Ou-Yang (2020) [44]	*	*	*	-	**	*	-	*	Good
Sharma (2018) [30]	*	*	*	-	-	*	*	*	Poor
Skoglund (2017) [49]	*	*	*	*	-	*	*	*	Poor
Sosnowski (2016) [31]	*	*	*	-	**	*	*	-	Good
Steinberg (2017) [32]	*	*	*	*	**	*	-	-	Poor
Van Dijck (2014) [33]	*	*	*	*	-	*	*	-	Poor
Wadia (2018) [34]	*	*	*	*	*	*	*	-	Good

Note: A study can be awarded a maximum of 1 star for each numbered item within the selection and exposure categories. A maximum of 2 stars can be given for comparability.
