# Supplementary material for: Comparative effects of transcatheter versus surgical pulmonary valve replacement: A systematic review and meta-analysis
Source: PLoS One. 2025 May 20;20(5):e0322041. doi: 10.1371/journal.pone.0322041 (PMC12091831; doi:10.1371/journal.pone.0322041)
Supplement: S10 Table — (PDF) [file pone.0322041.s010.pdf]

S10 Table. Domains of potential confounders observed in the studies.

Domains	Example of confounding variables
Patient demographics	Age, body mass index, body surface area, race, sex, weight
Prior procedure	Endovascular transcatheter pulmonary valve replacement, long-term anticoagulation, open heart surgery, right atrium or ventricle to pulmonary artery, right ventricular to pulmonary artery conduit, transapical transcatheter pulmonary valve replacement
Comorbidities	New York Heart Association functional class, rheumatic heart disease, aortic regurgitation, aortic stenosis, atrial arrhythmia, coronary artery disease, coronary heart failure, diabetes mellitus, history of gastrointestinal condition, history of hematologic condition, history of infective endocarditis, history of malignancy, history of metabolic condition, history of neurologic condition, history of renal condition, history of respiratory condition, hypertension, known genetic syndrome, left ventricular dysfunction, non-cardiac congenital anomaly, obesity, obstructive sleep apnea, right ventricular dysfunction, seizure disorder, smoker, substance or drug abuse, ventricular arrhythmia, ventricular septal defect
Primary diagnosis	Other congenital abnormality of pulmonary valve, pulmonary annulus diameter, pulmonary regurgitation, pulmonary regurgitant grade, pulmonary stenosis, tetralogy of Fallot, tricuspid regurgitant grade
