# Supplementary figures and images for: Comparative effects of transcatheter versus surgical pulmonary valve replacement: A systematic review and meta-analysis
Source: PLoS One. 2025 May 20;20(5):e0322041. doi: 10.1371/journal.pone.0322041 (PMC12091831; doi:10.1371/journal.pone.0322041)

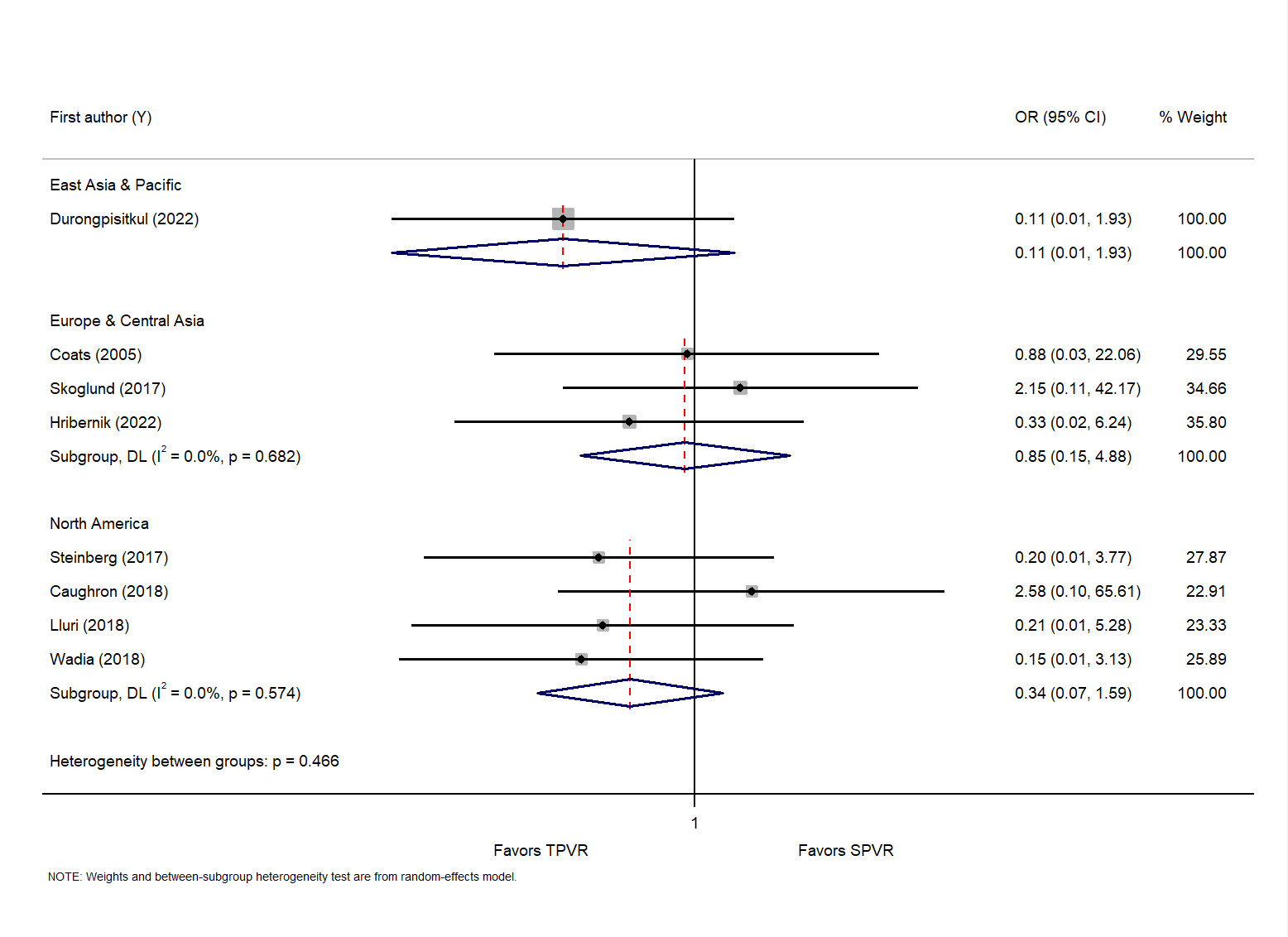

Supplement: S1 Fig — OR, odds ratio. (TIF) [file pone.0322041.s011.tif]

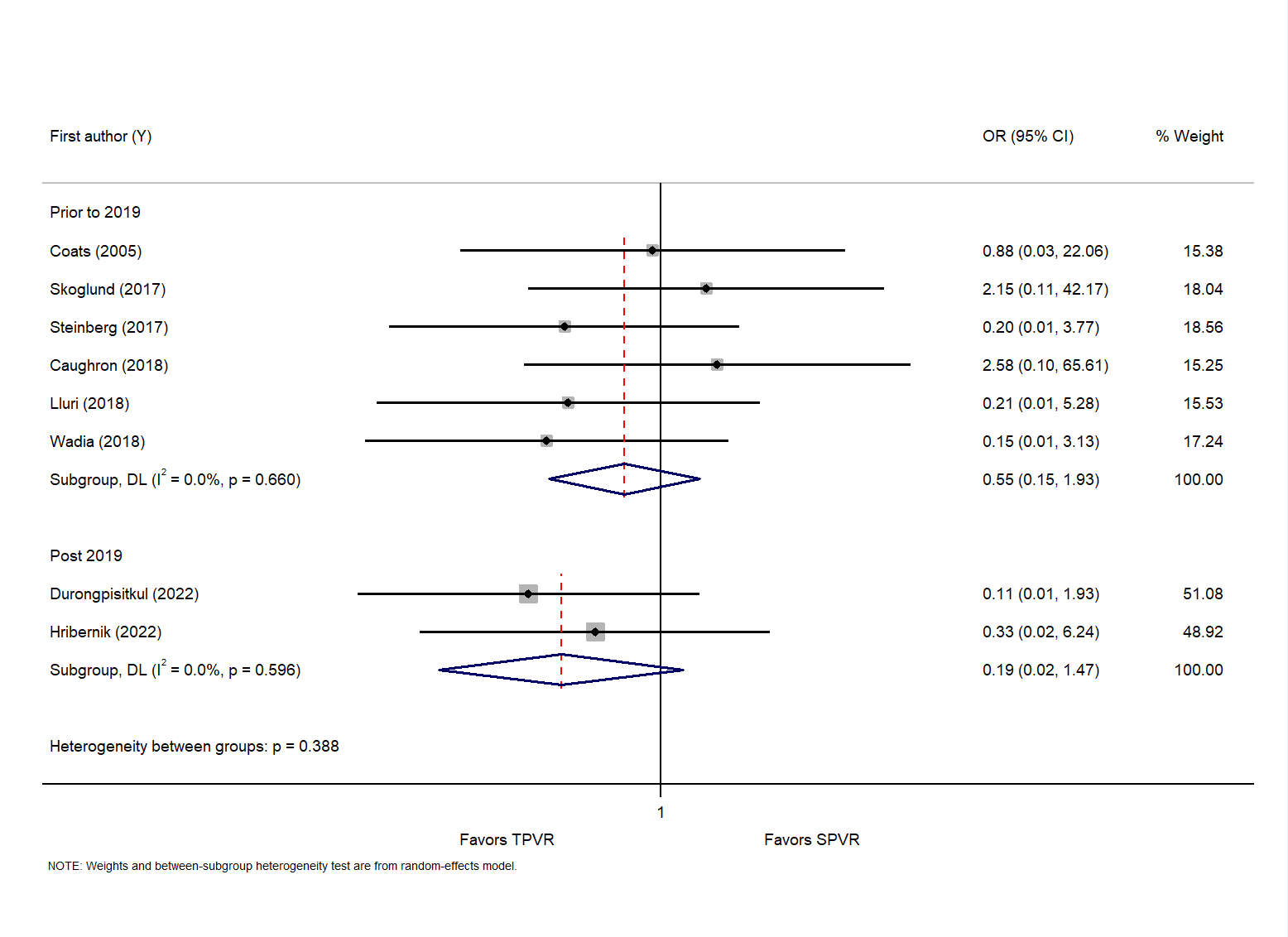

Supplement: S3 Fig — OR, odds ratio. (TIF) [file pone.0322041.s013.tif]

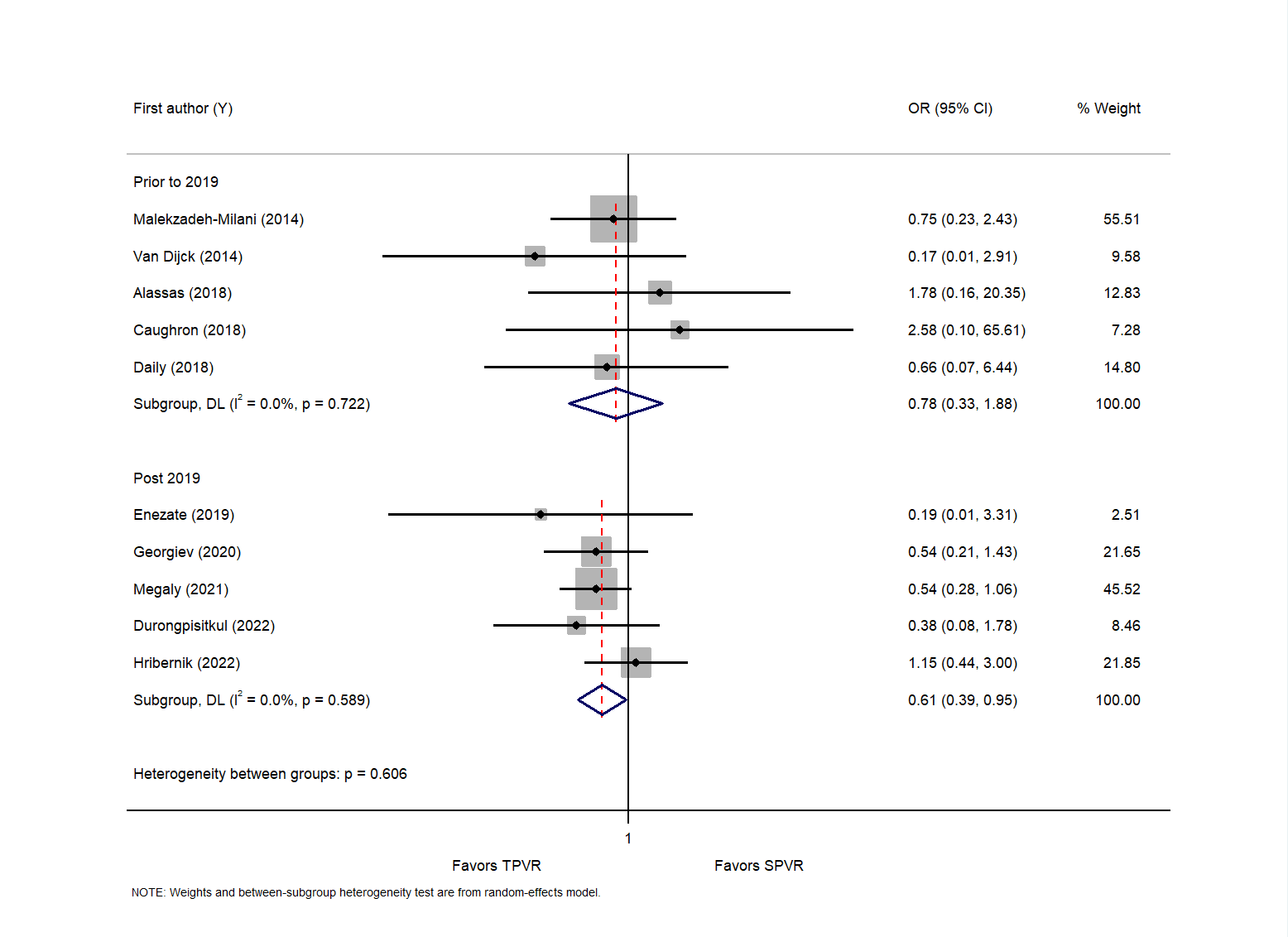

Supplement: S4 Fig — OR, odds ratio. (TIF) [file pone.0322041.s014.tif]

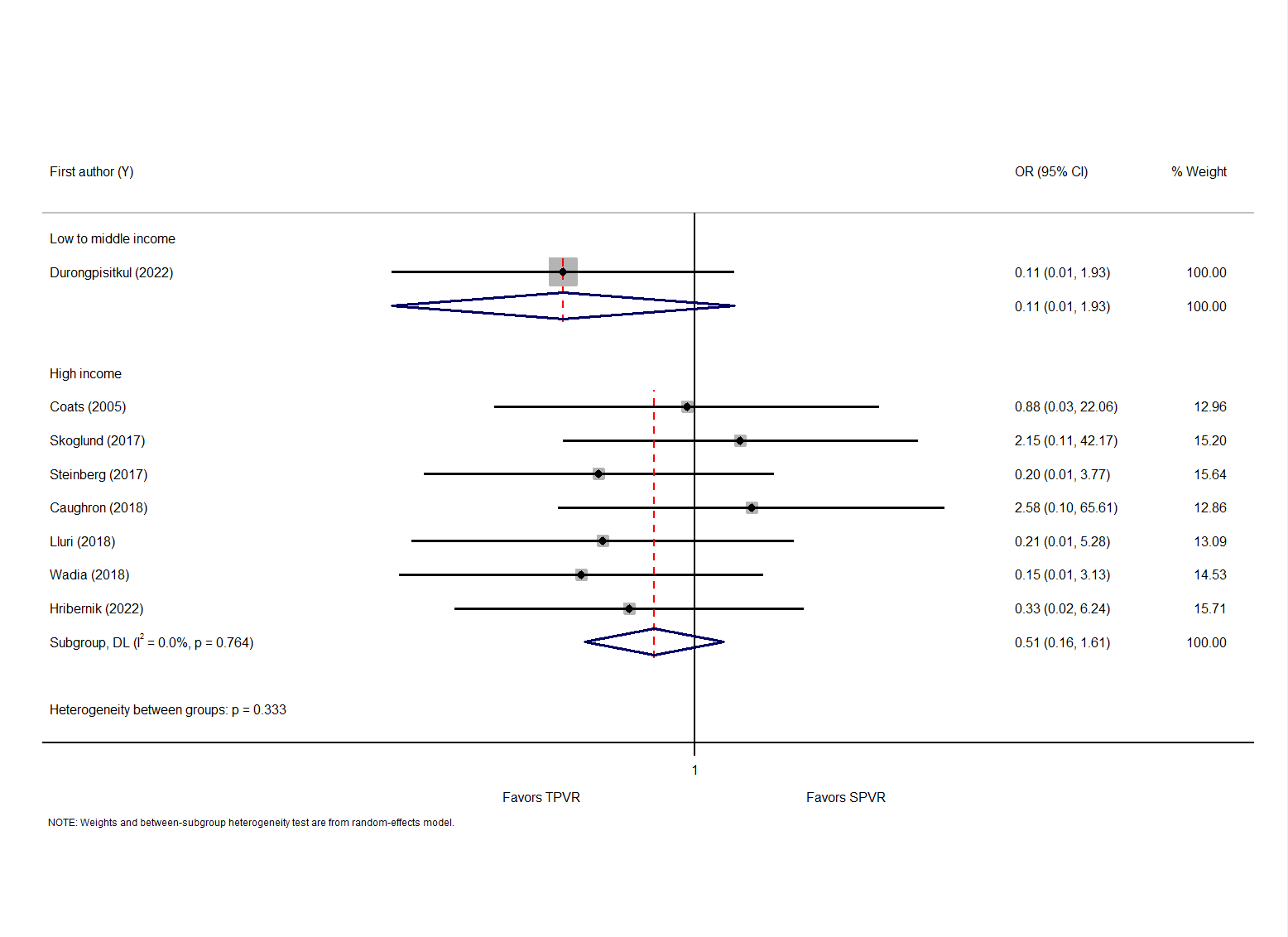

Supplement: S5 Fig — OR, odds ratio. (TIF) [file pone.0322041.s015.tif]

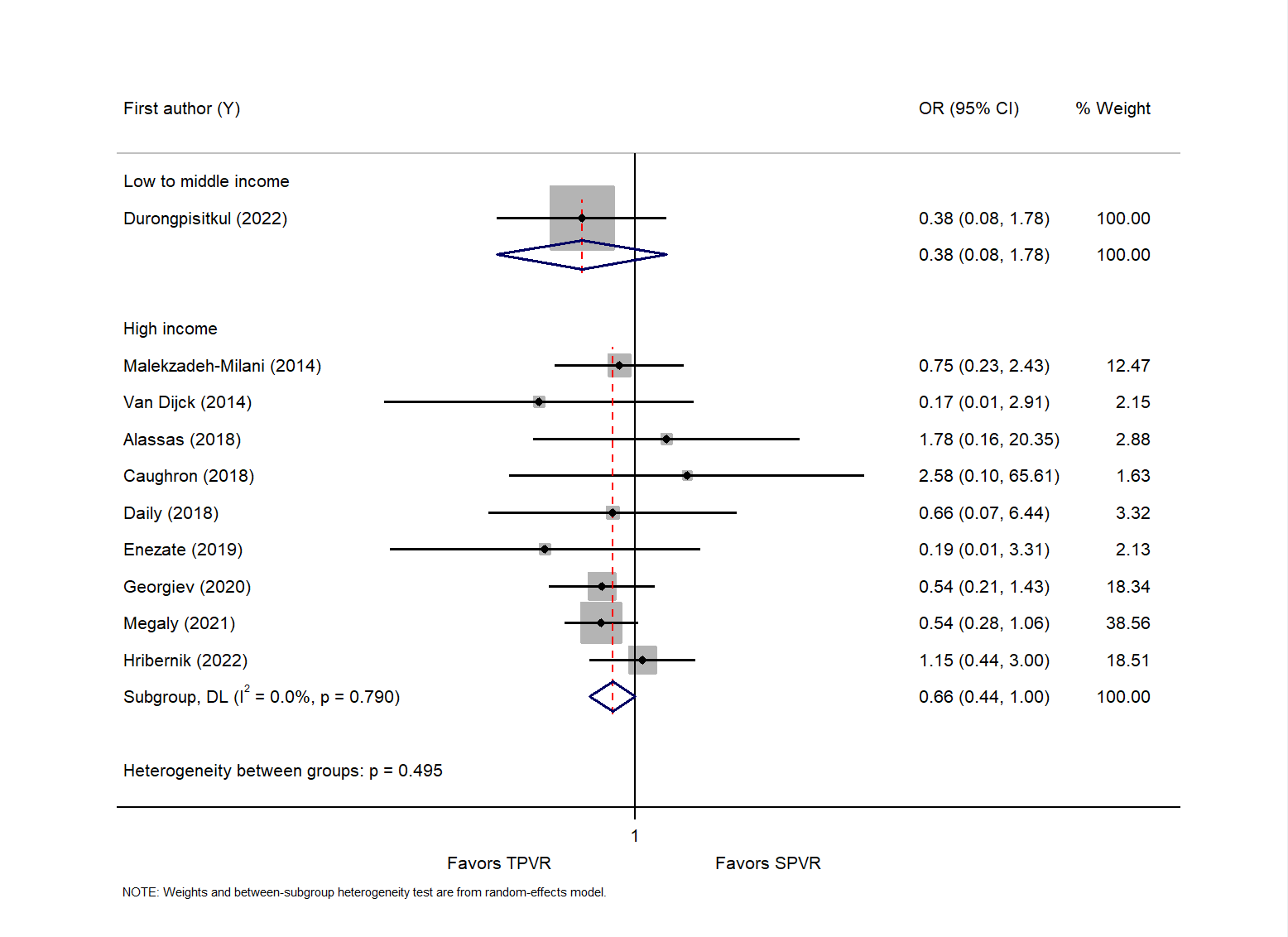

Supplement: S6 Fig — OR, odds ratio. (TIF) [file pone.0322041.s016.tif]

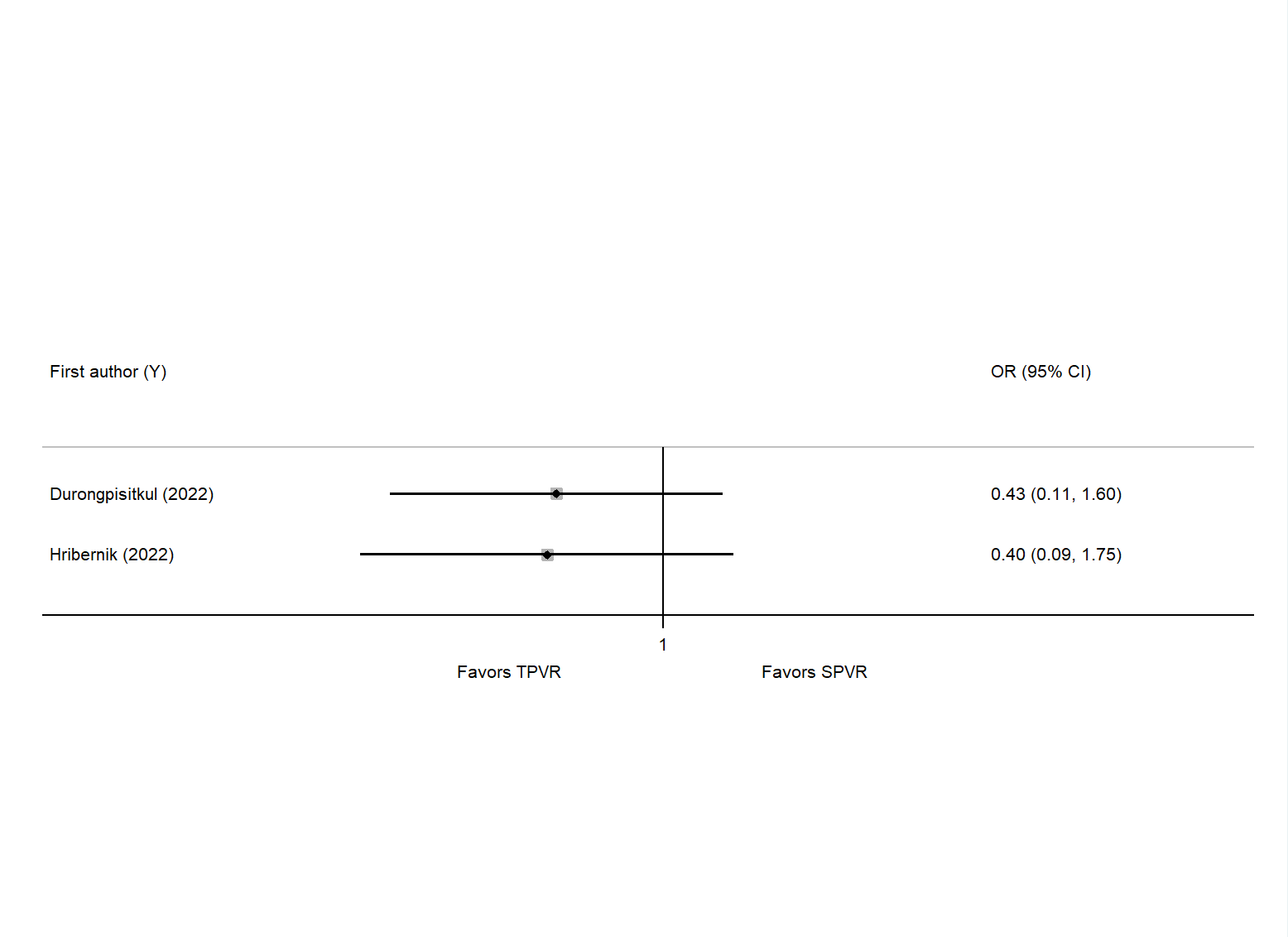

Supplement: S7 Fig — OR, odds ratio. (TIF) [file pone.0322041.s017.tif]

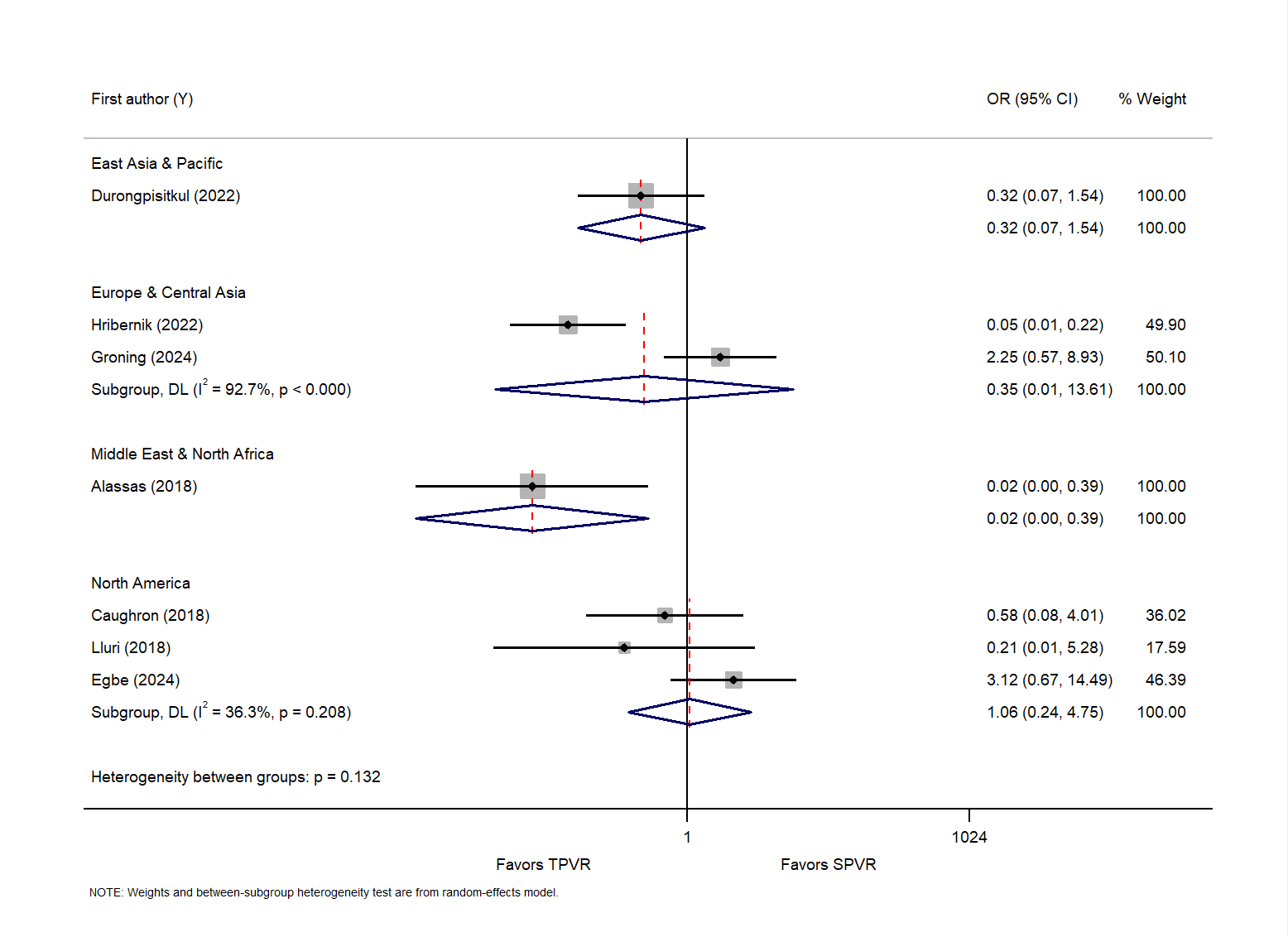

Supplement: S8 Fig — OR, odds ratio. (TIF) [file pone.0322041.s018.tif]

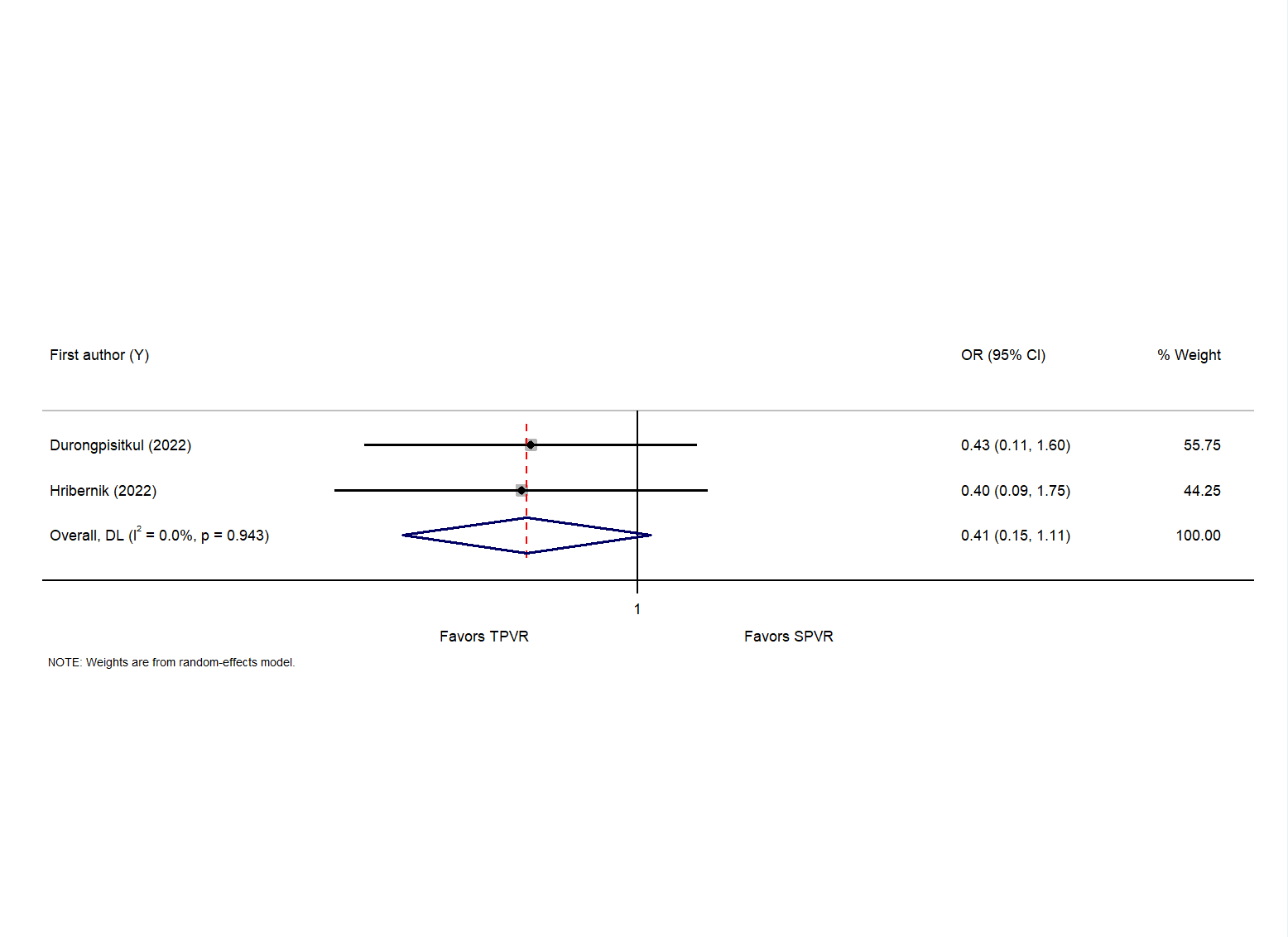

Supplement: S9 Fig — OR, odds ratio. (TIF) [file pone.0322041.s019.tif]

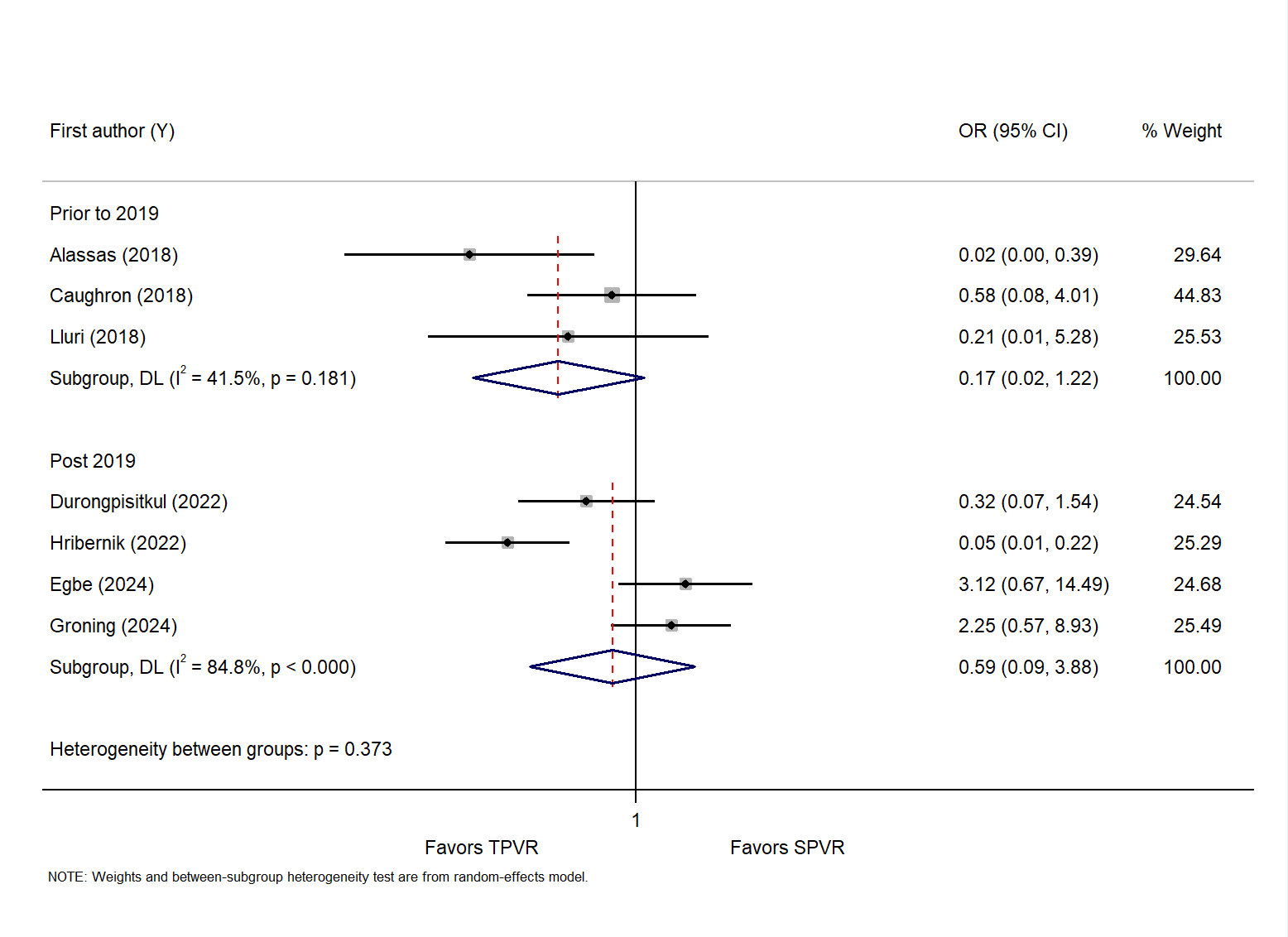

Supplement: S10 Fig — OR, odds ratio. (TIF) [file pone.0322041.s020.tif]

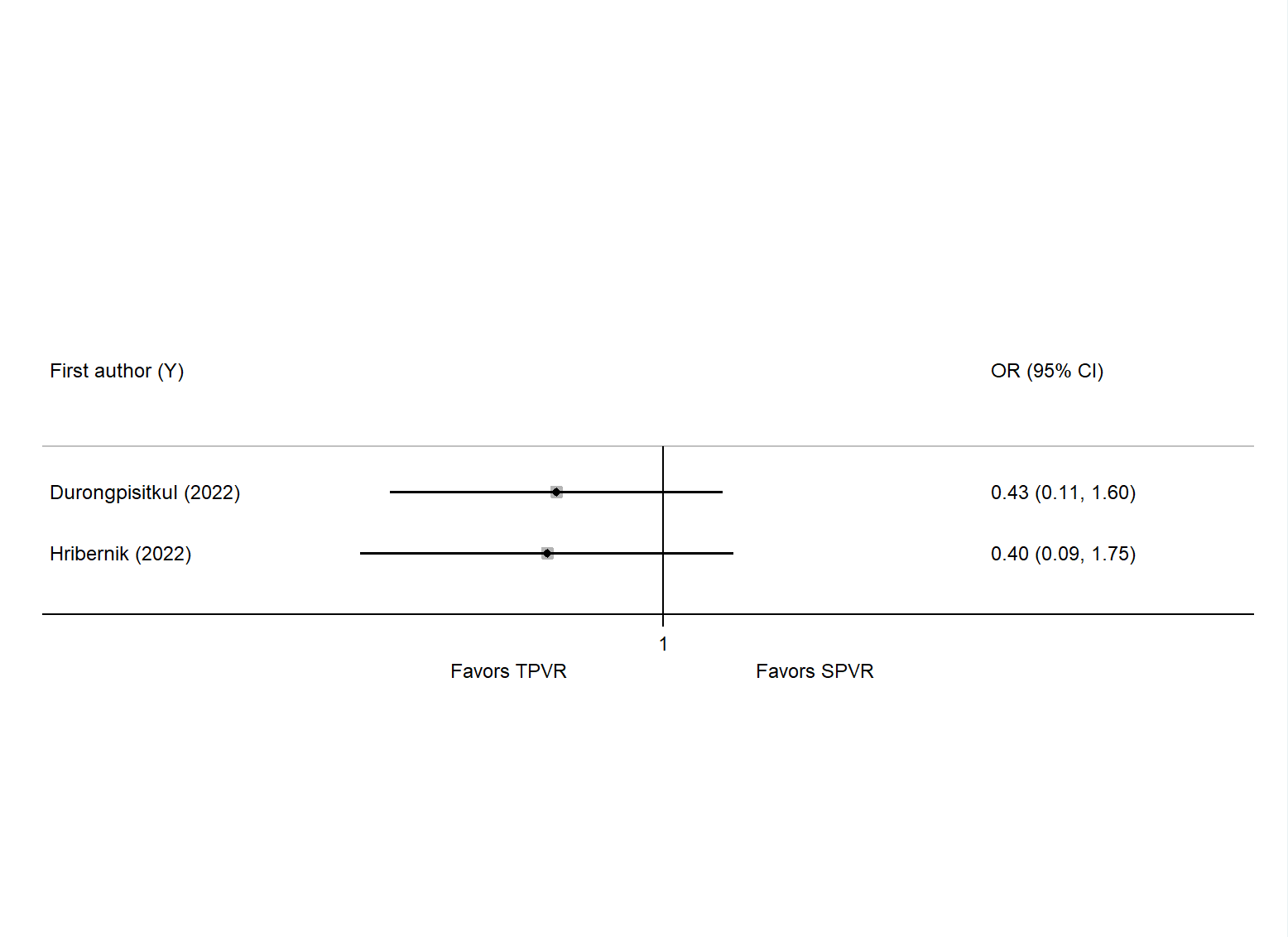

Supplement: S11 Fig — OR, odds ratio. (TIF) [file pone.0322041.s021.tif]

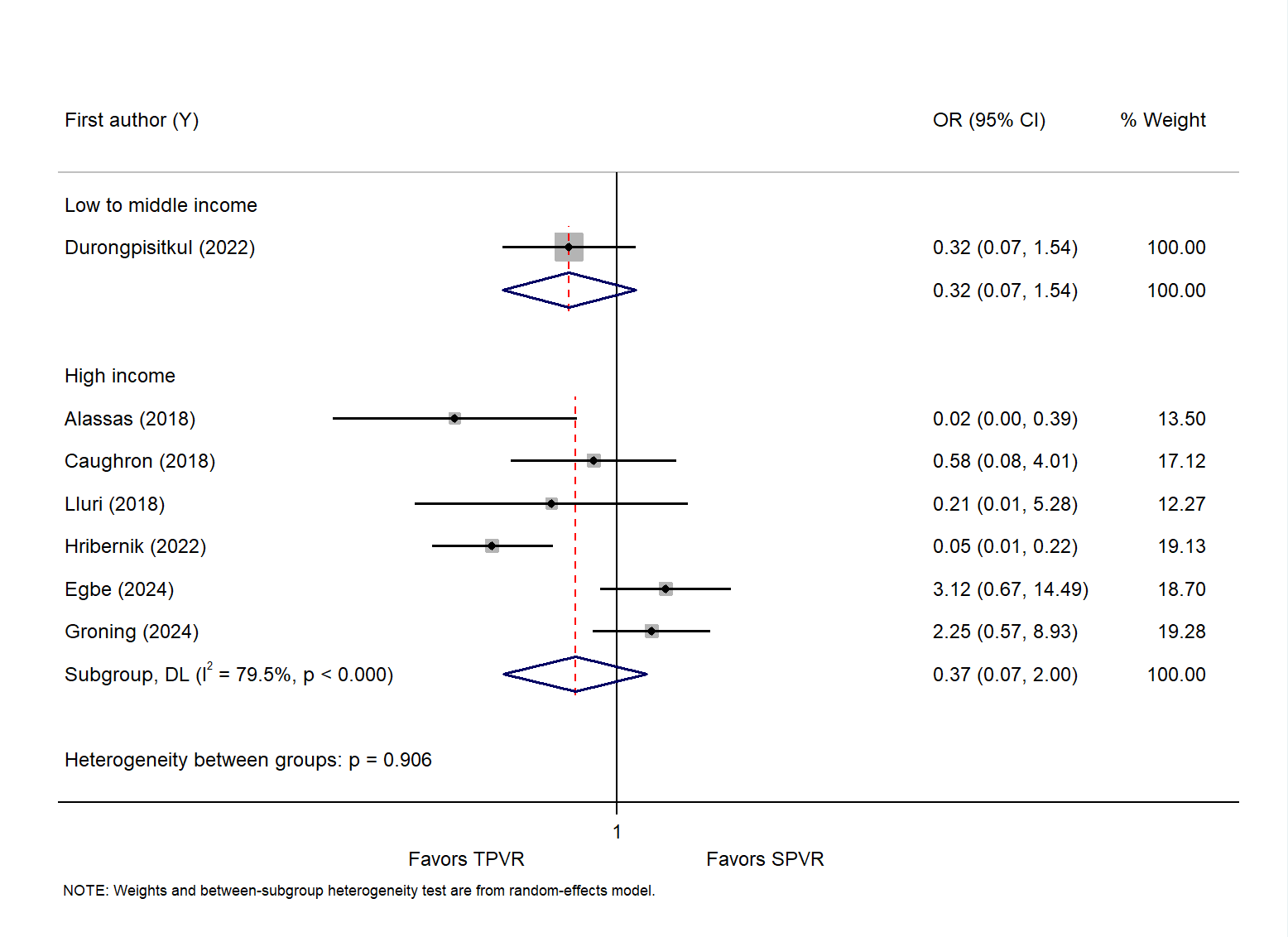

Supplement: S12 Fig — OR, odds ratio. (TIF) [file pone.0322041.s022.tif]

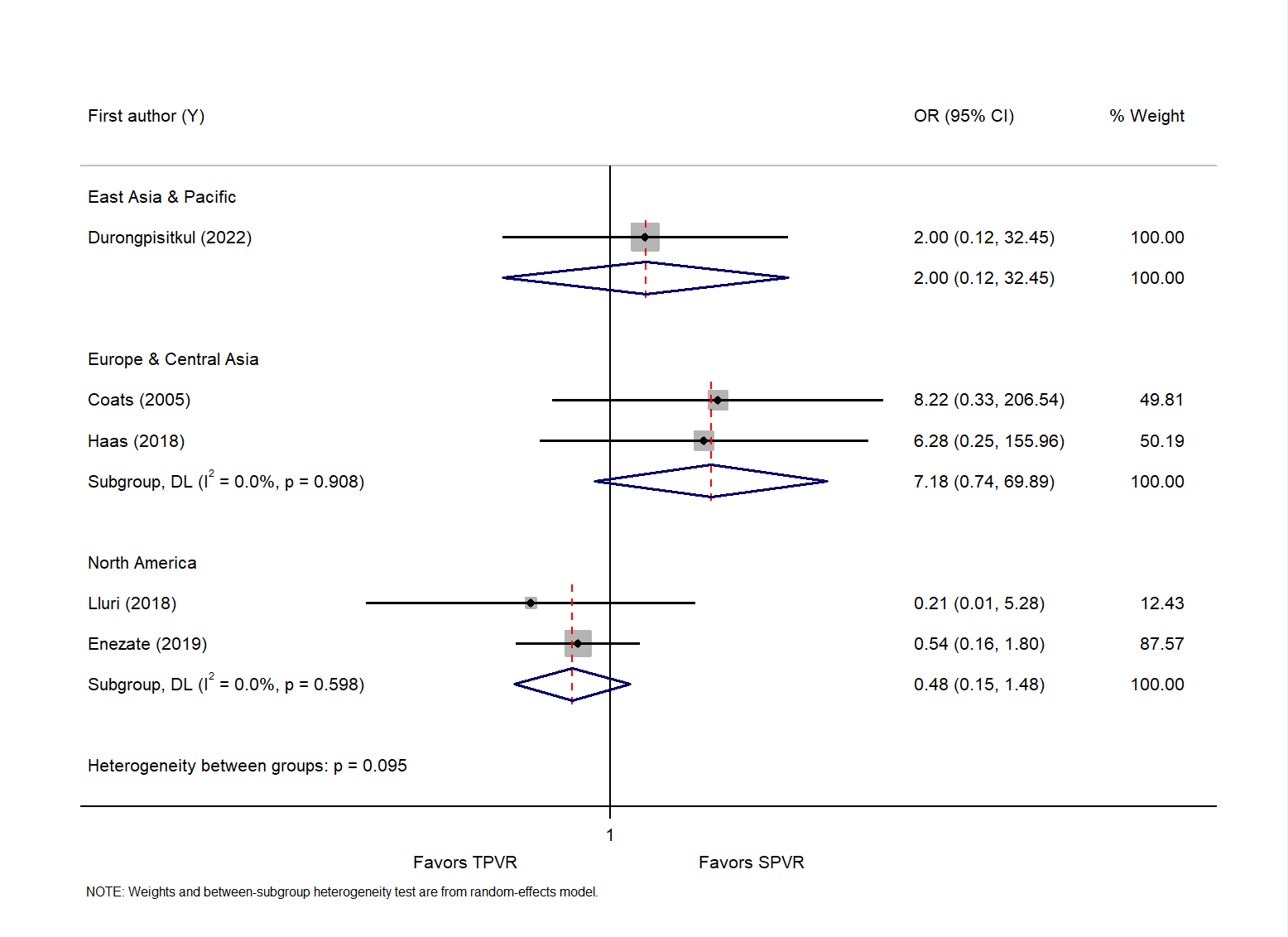

Supplement: S13 Fig — OR, odds ratio. (TIF) [file pone.0322041.s023.tif]

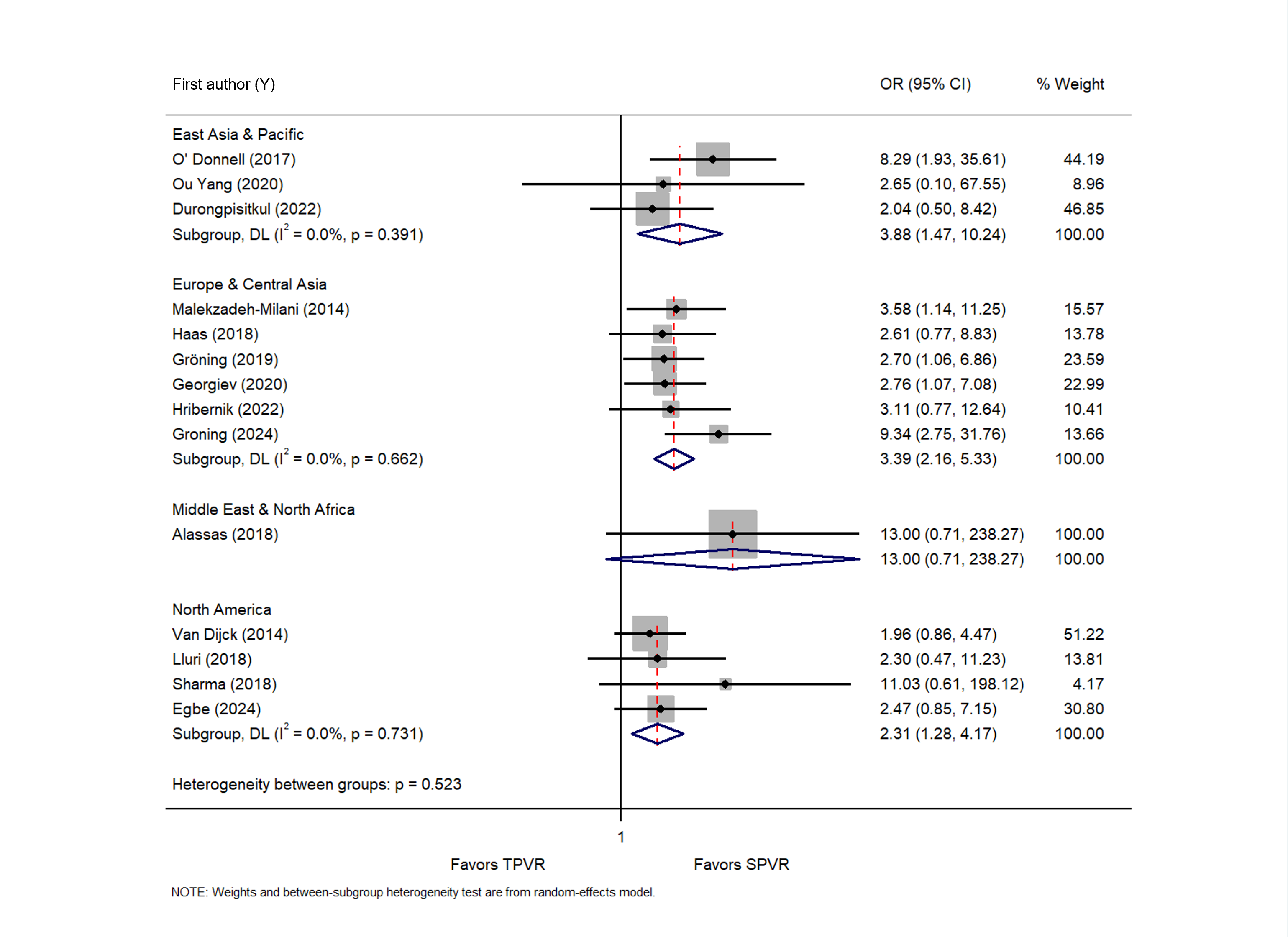

Supplement: S14 Fig — OR, odds ratio. (TIF) [file pone.0322041.s024.tif]

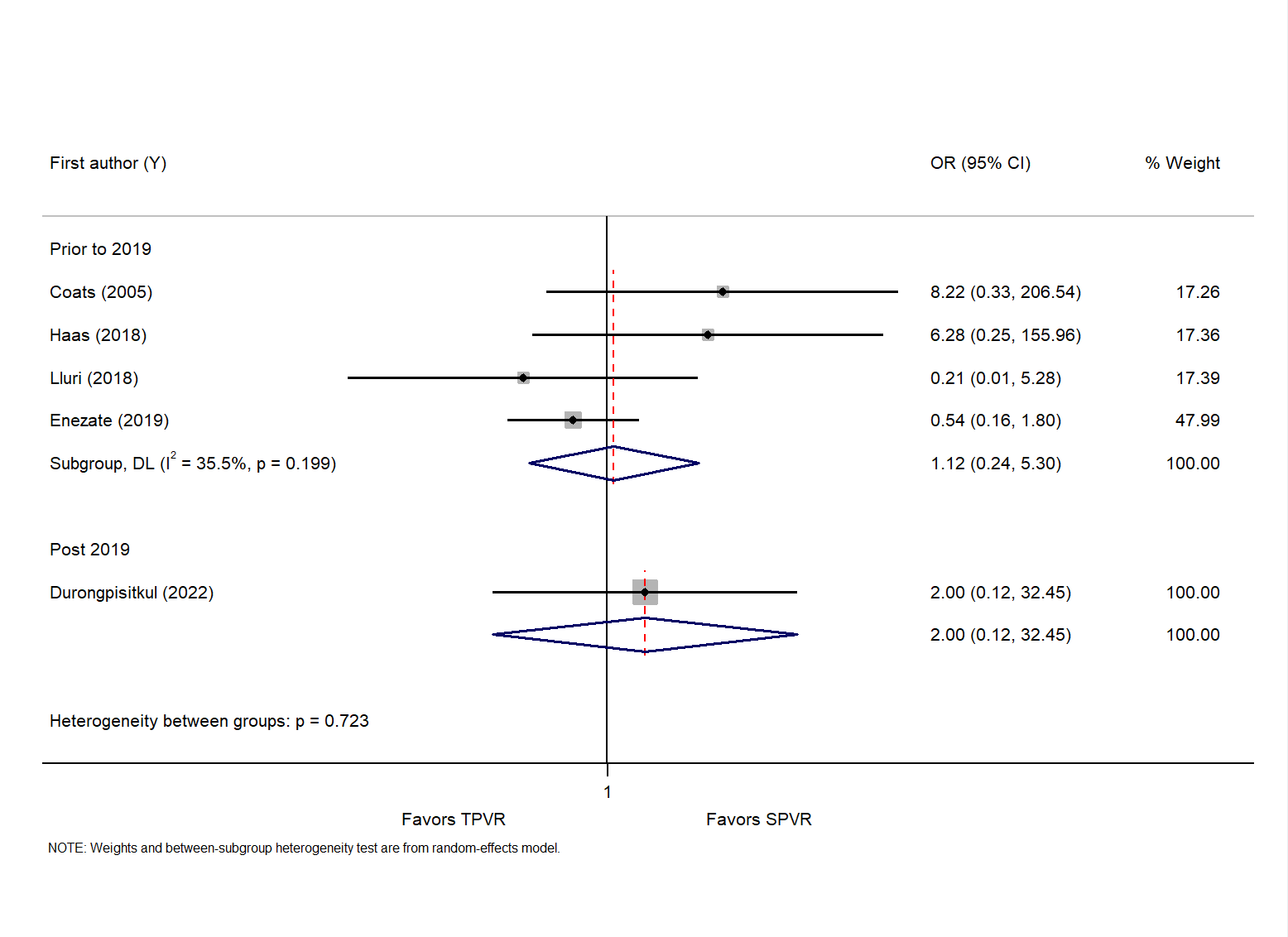

Supplement: S15 Fig — OR, odds ratio. (TIF) [file pone.0322041.s025.tif]

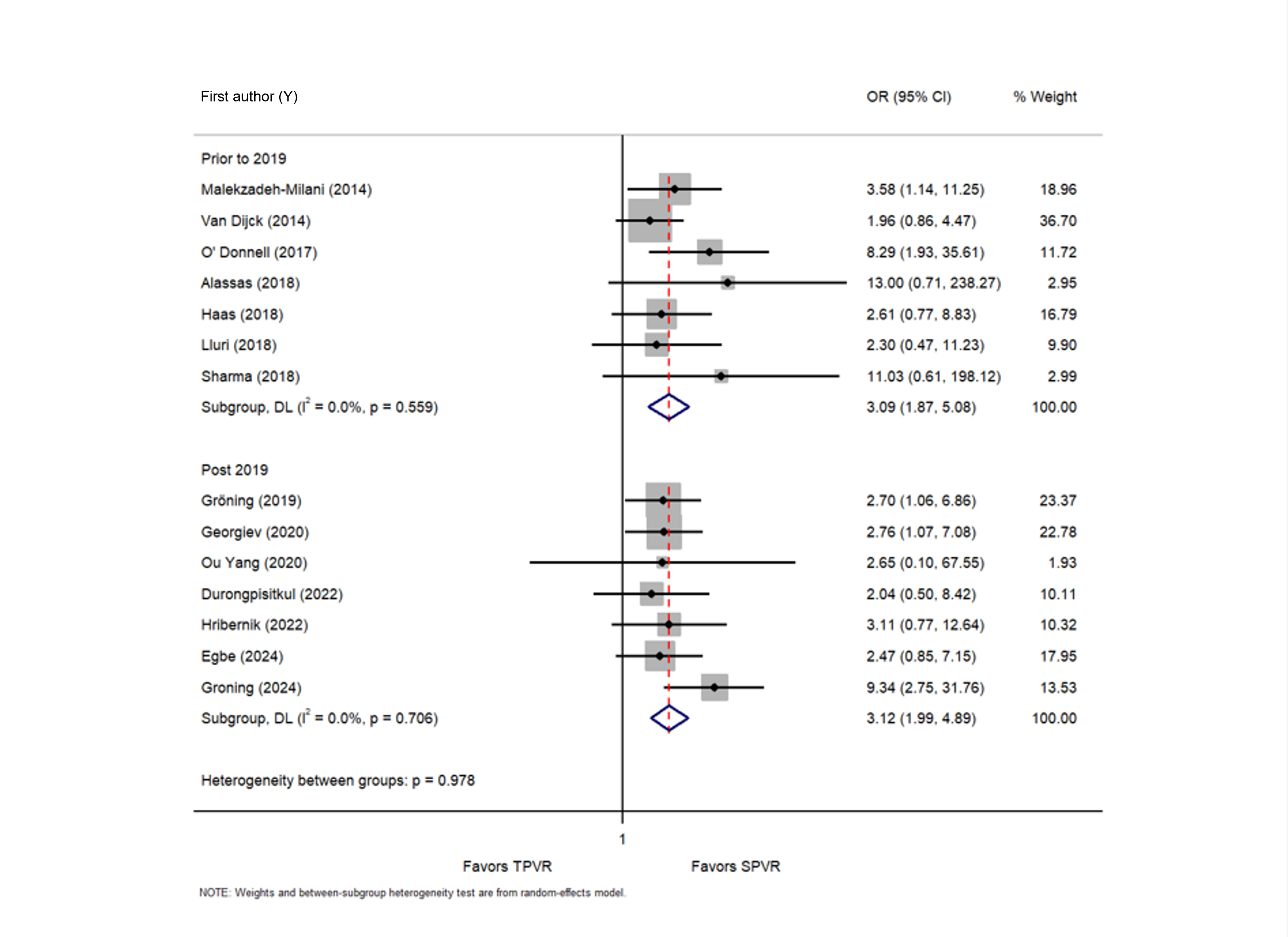

Supplement: S16 Fig — OR, odds ratio. (TIF) [file pone.0322041.s026.tif]

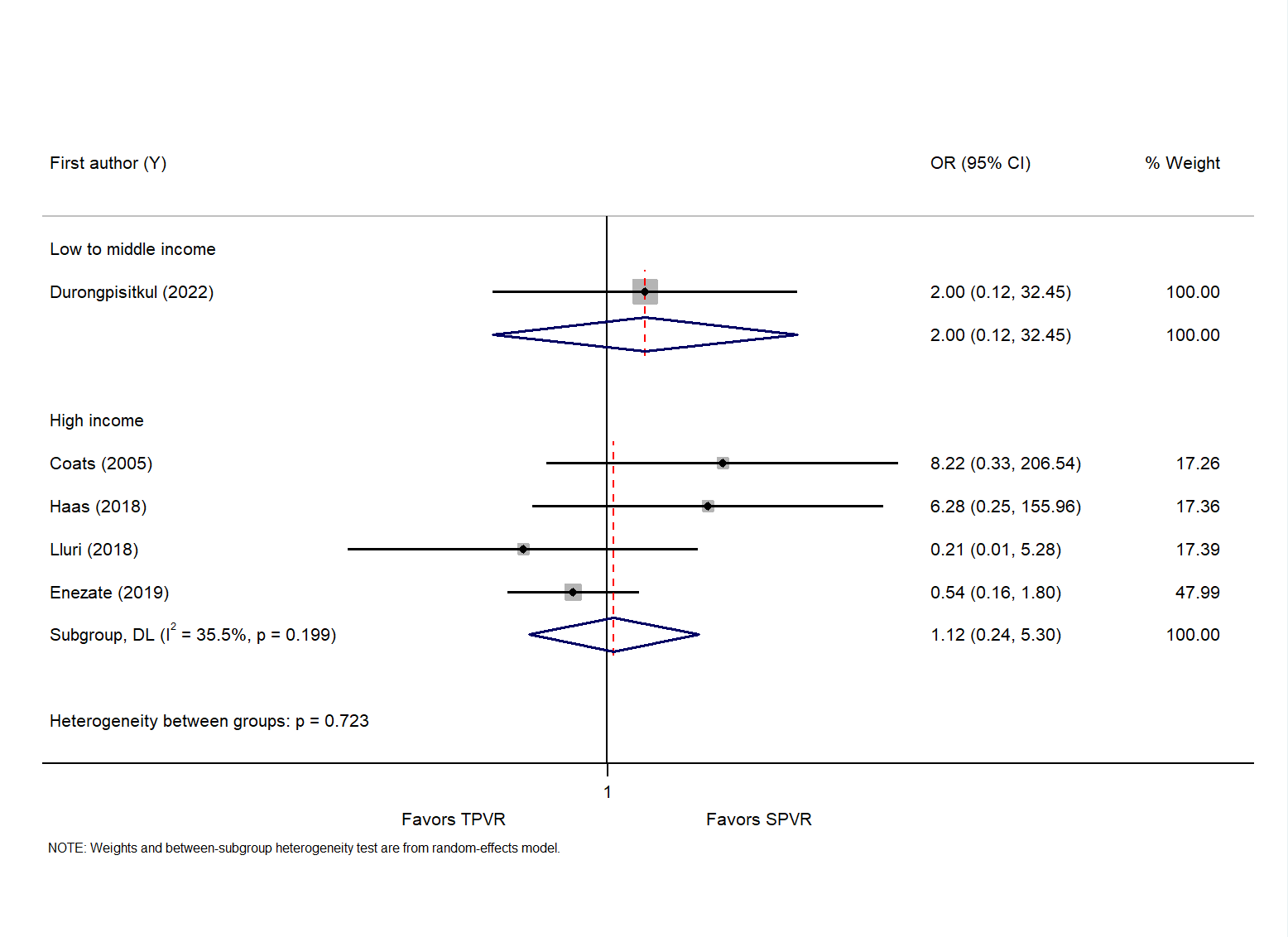

Supplement: S17 Fig — OR, odds ratio. (TIF) [file pone.0322041.s027.tif]

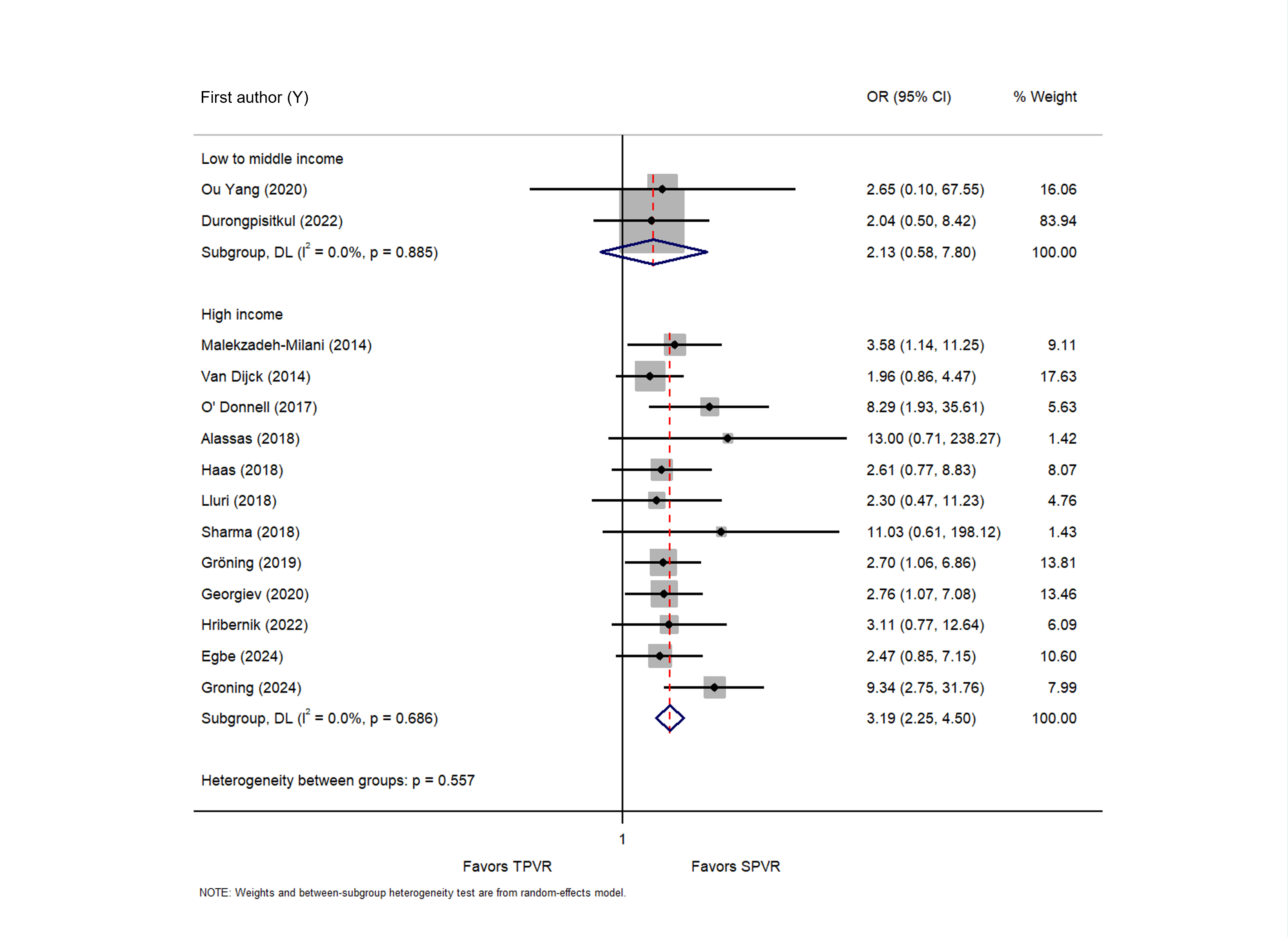

Supplement: S18 Fig — OR, odds ratio. (TIF) [file pone.0322041.s028.tif]

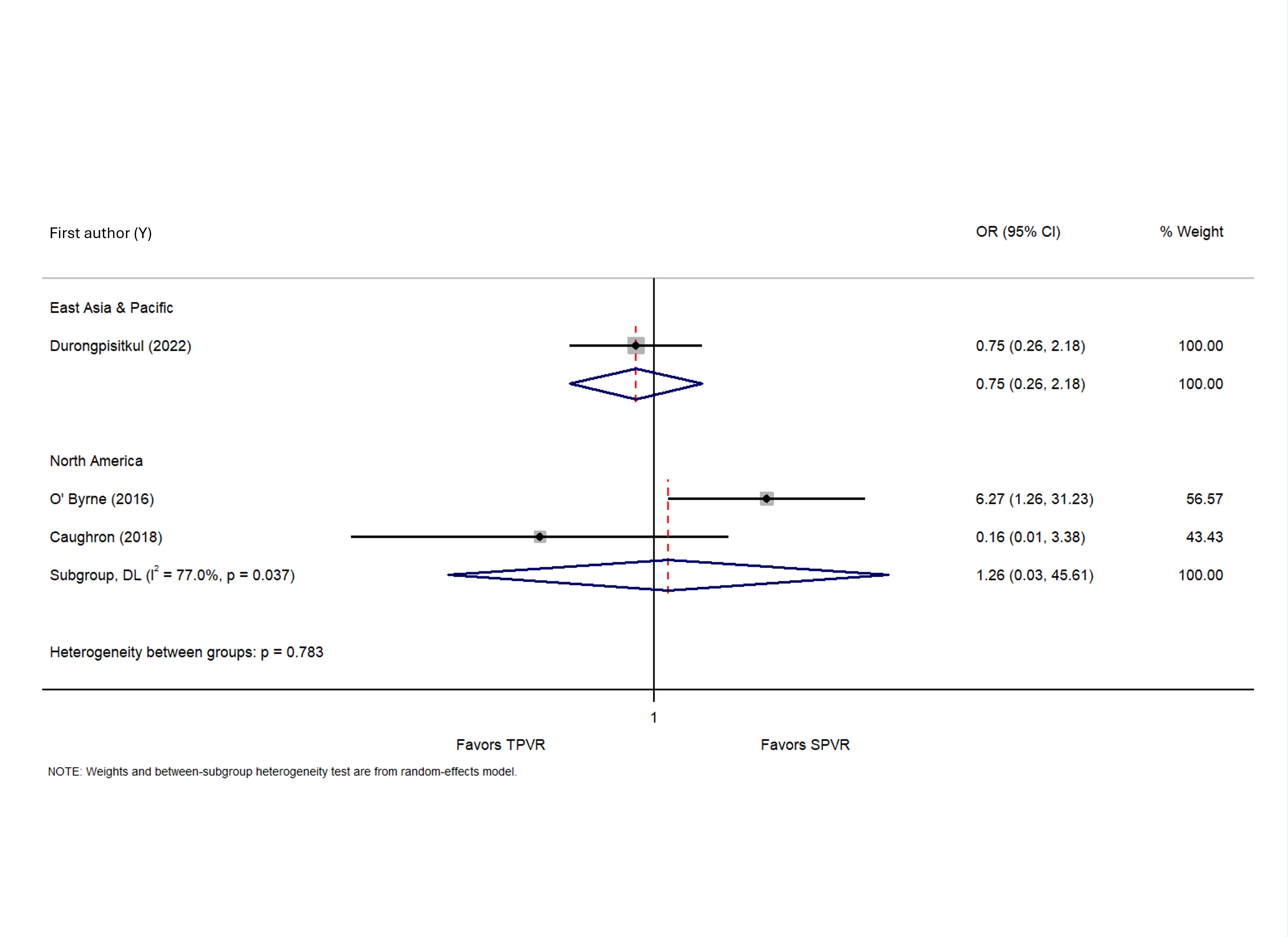

Supplement: S19 Fig — OR, odds ratio. (TIF) [file pone.0322041.s029.tif]

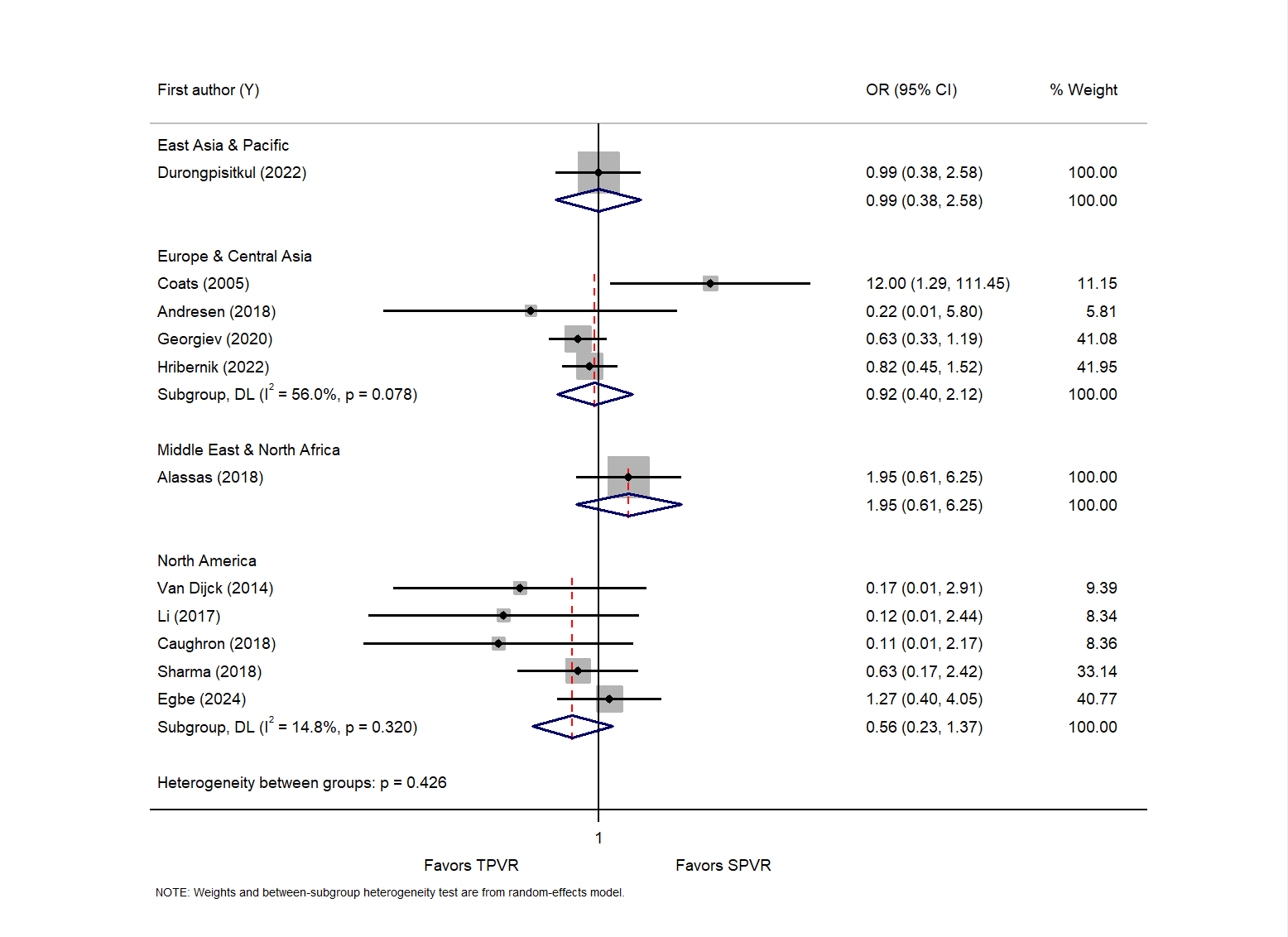

Supplement: S20 Fig — OR, odds ratio. (TIF) [file pone.0322041.s030.tif]

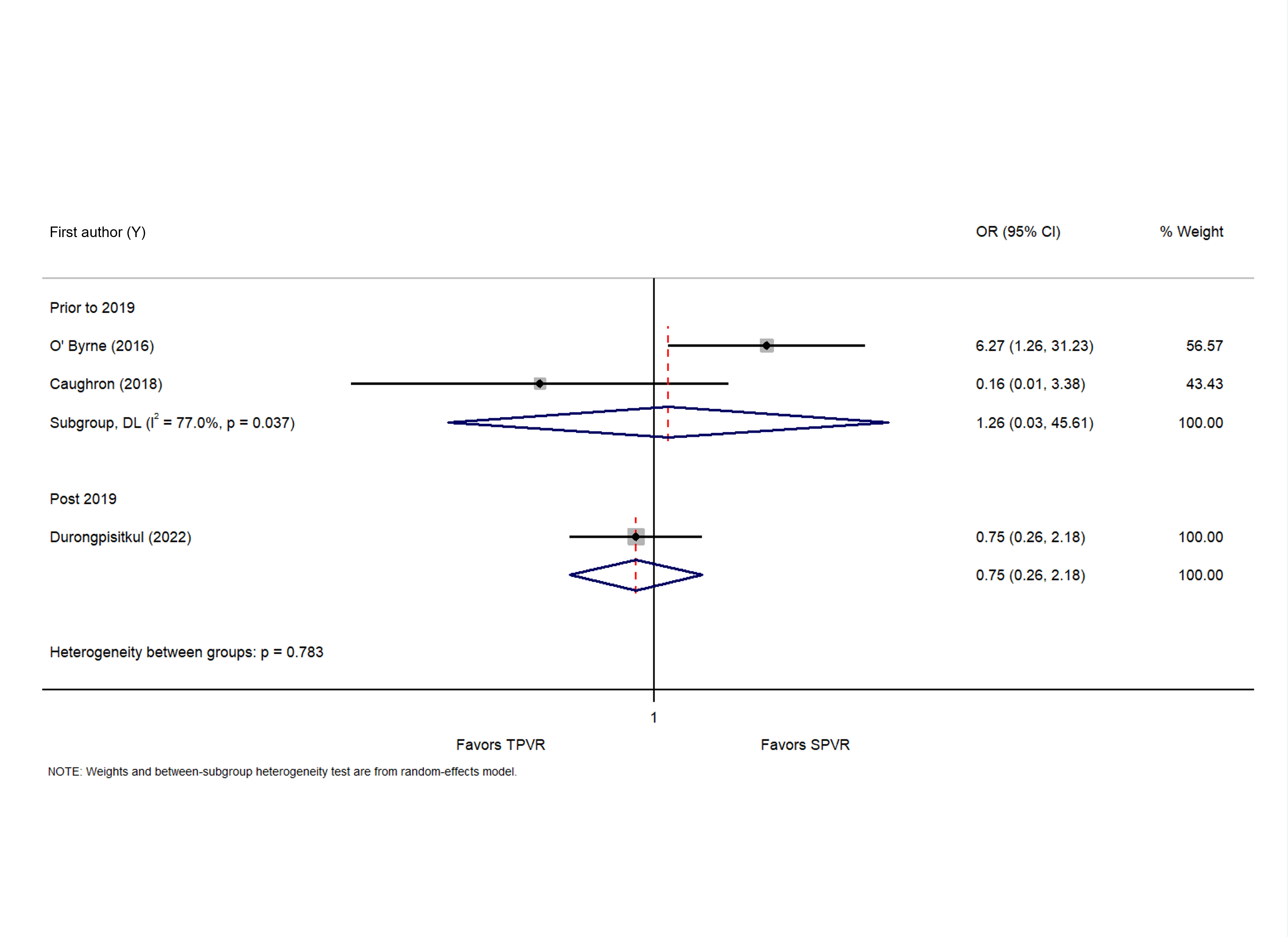

Supplement: S21 Fig — OR, odds ratio. (TIF) [file pone.0322041.s031.tif]

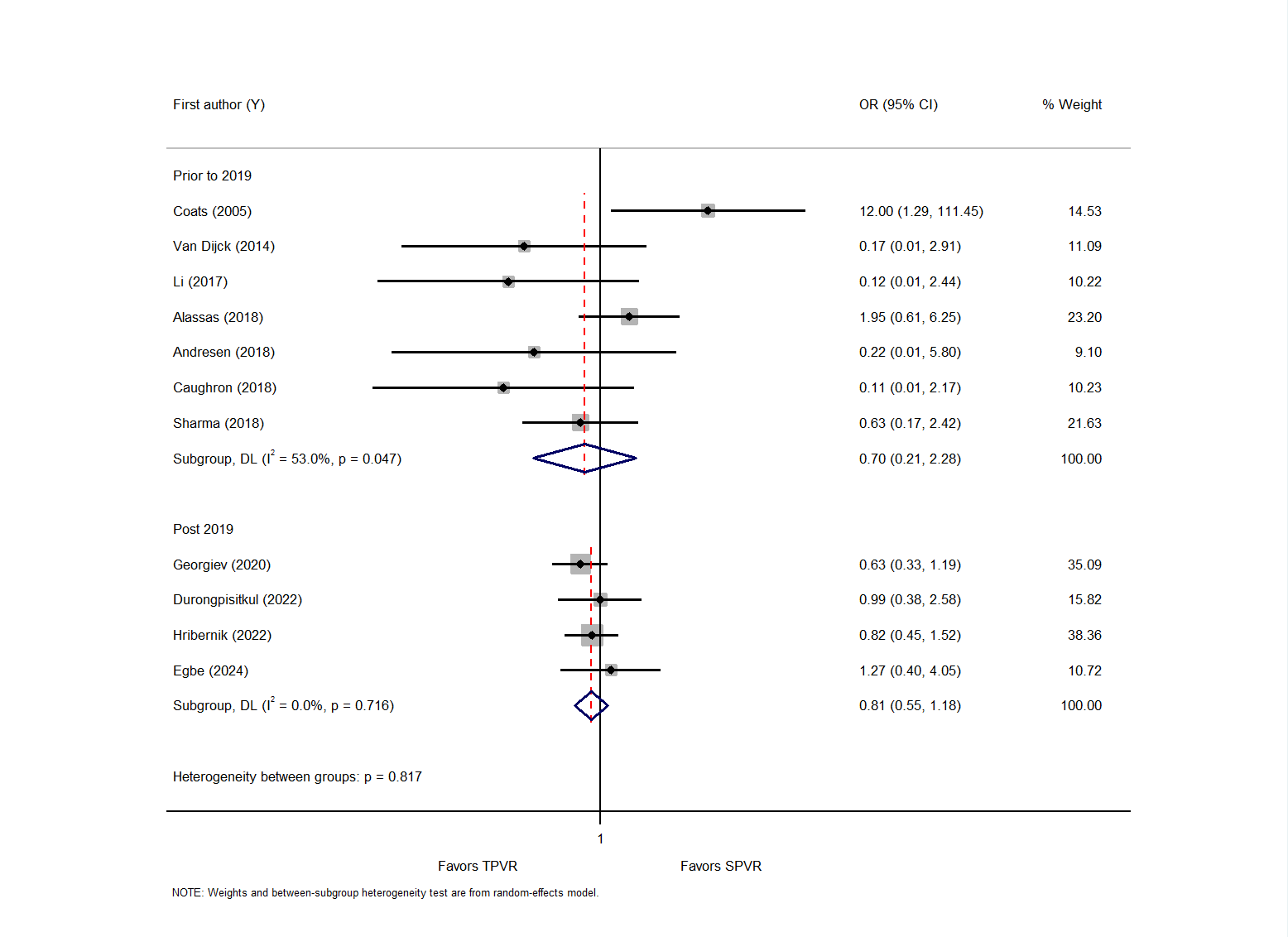

Supplement: S22 Fig — OR, odds ratio. (TIF) [file pone.0322041.s032.tif]

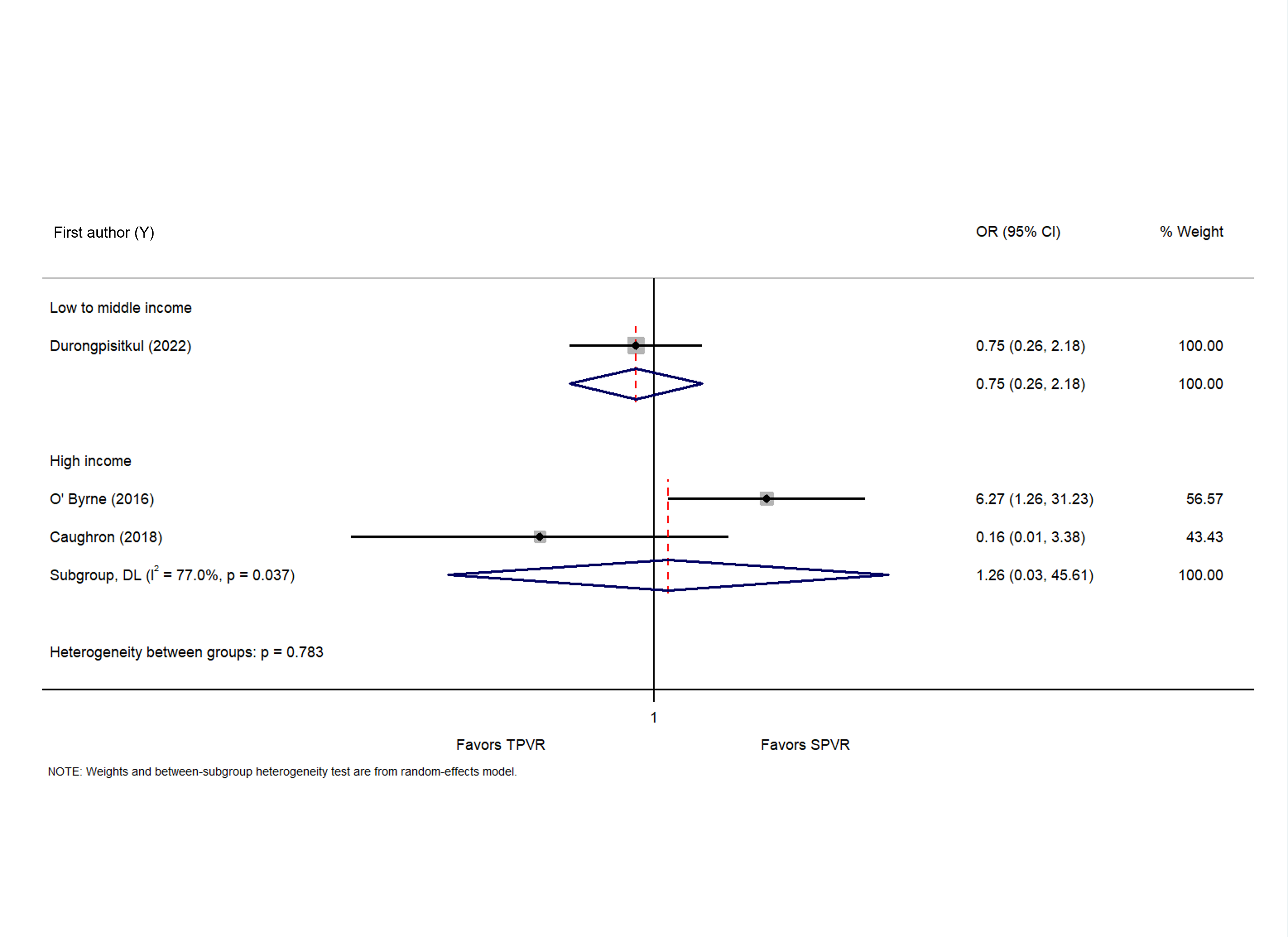

Supplement: S23 Fig — OR, odds ratio. (TIF) [file pone.0322041.s033.tif]

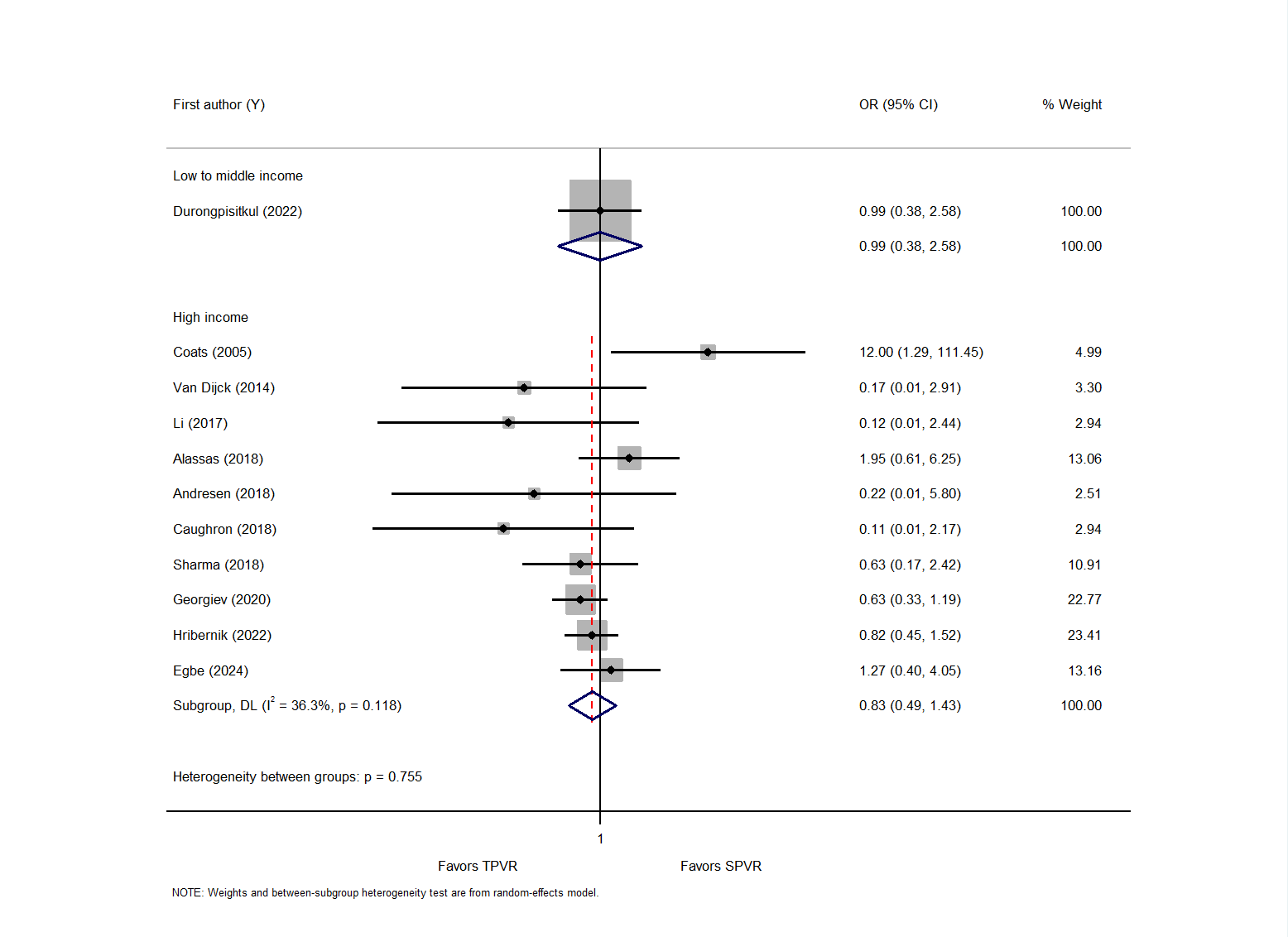

Supplement: S24 Fig — OR, odds ratio. (TIF) [file pone.0322041.s034.tif]

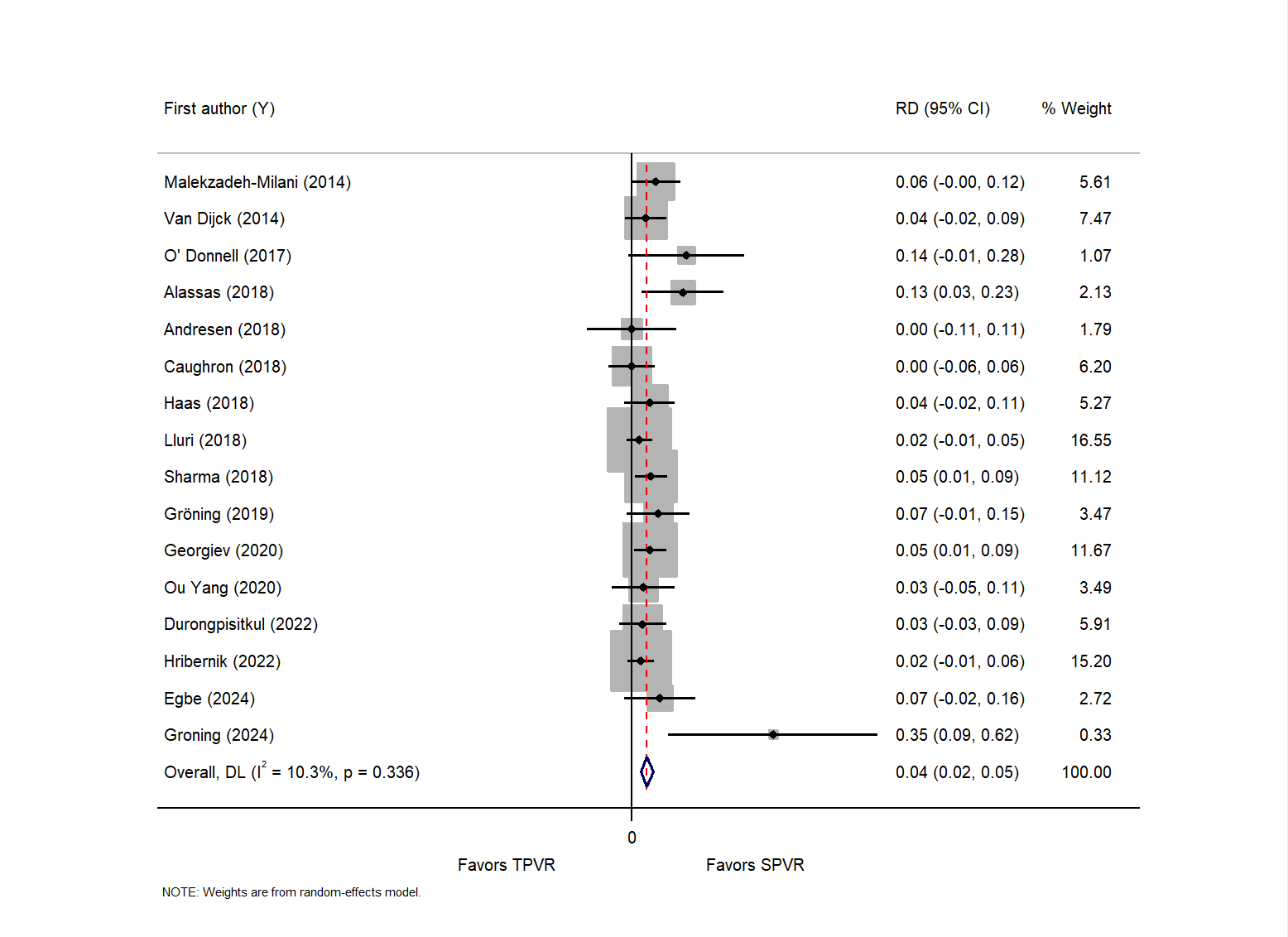

Supplement: S25 Fig — RD, risk difference. (TIF) [file pone.0322041.s035.tif]

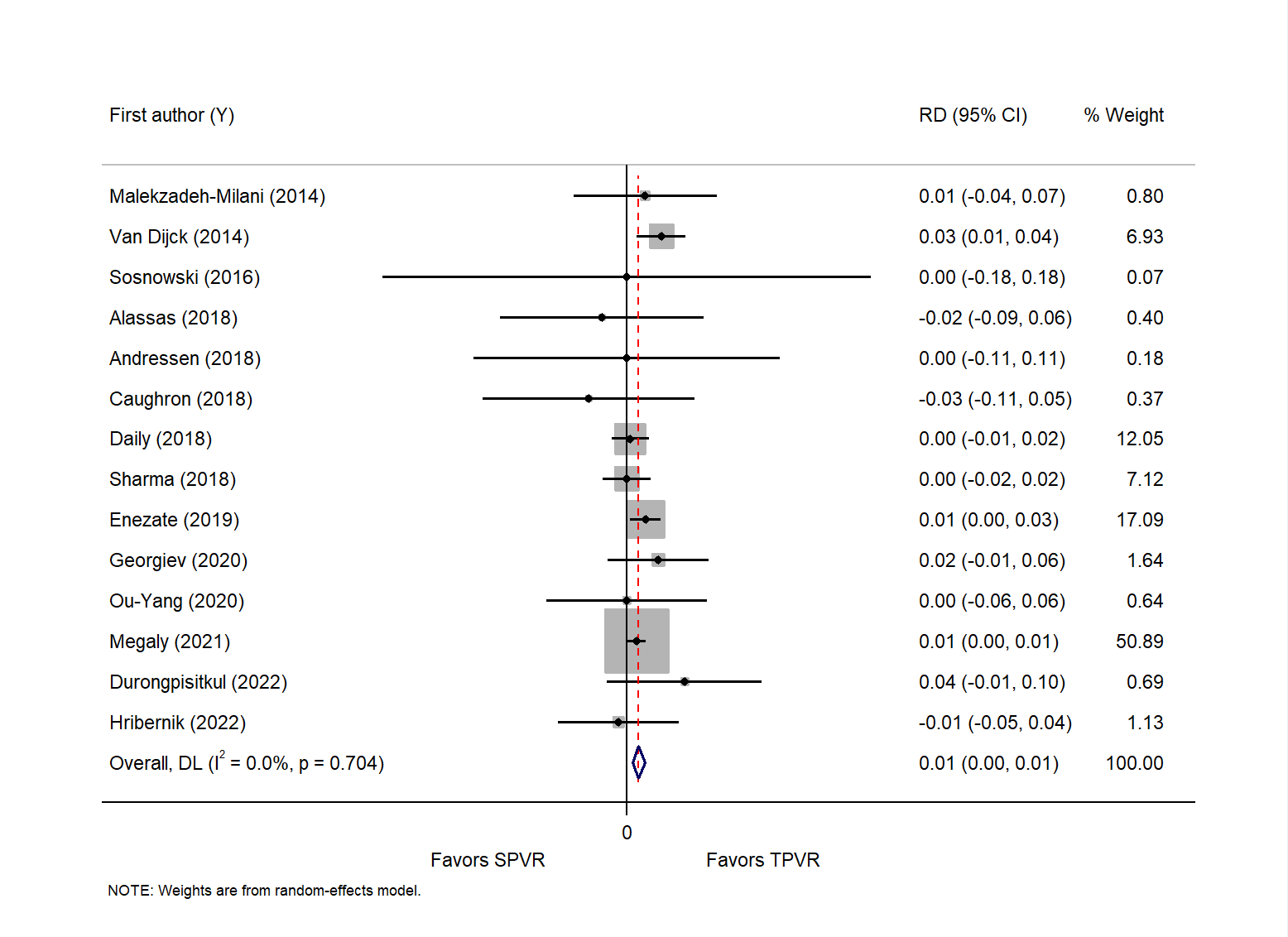

Supplement: S26 Fig — RD, risk difference. (TIF) [file pone.0322041.s036.tif]

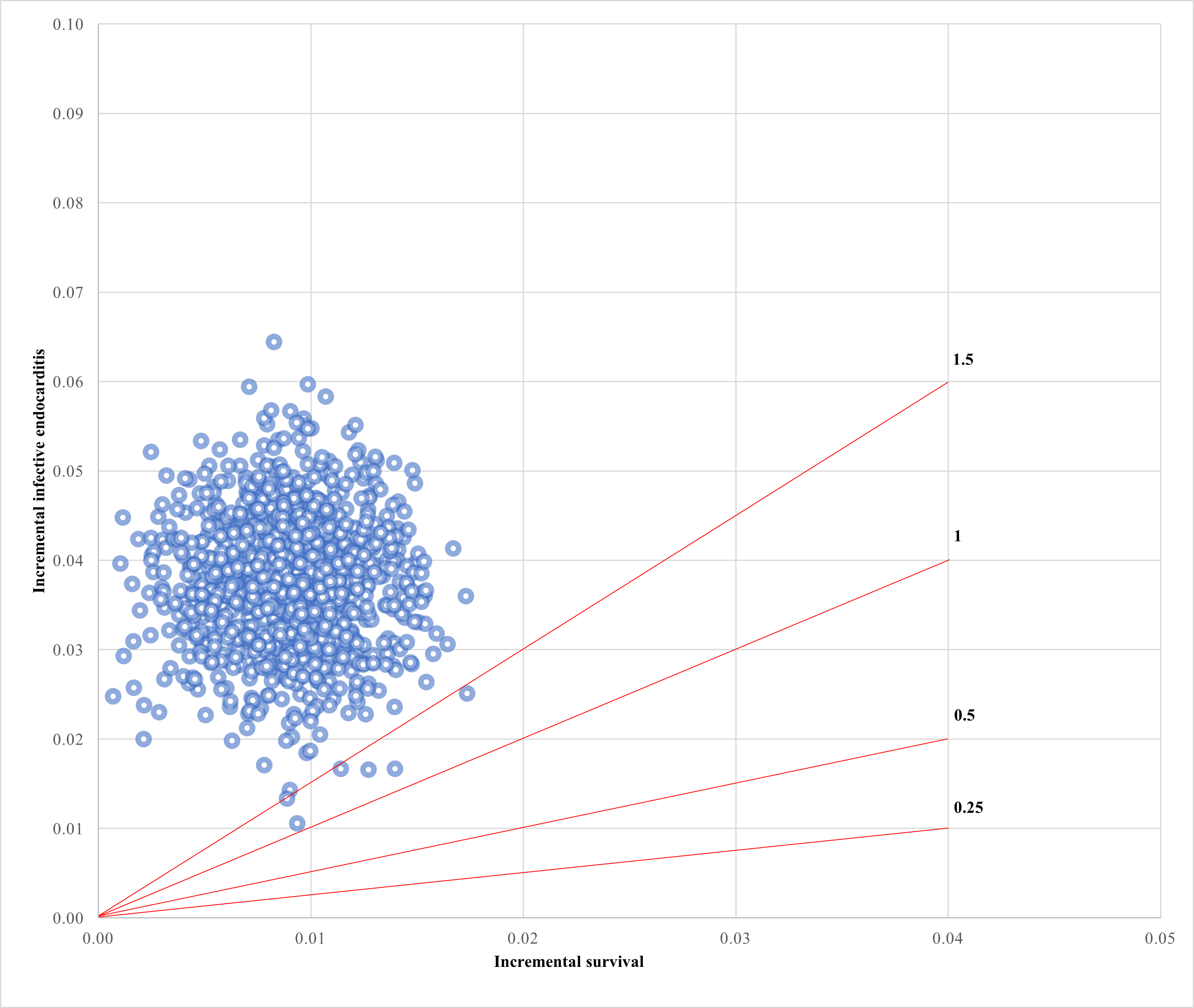

Supplement: S27 Fig — (TIF) [file pone.0322041.s037.tif]
